# Exposure to hate in online and traditional media: A systematic review and meta‐analysis of the impact of this exposure on individuals and communities

**DOI:** 10.1002/cl2.70018

**Published:** 2025-01-16

**Authors:** Pablo Madriaza, Ghayda Hassan, Sébastien Brouillette‐Alarie, Aoudou Njingouo Mounchingam, Loïc Durocher‐Corfa, Eugene Borokhovski, David Pickup, Sabrina Paillé

**Affiliations:** ^1^ Department of Psychoeducation and Social Work Université du Québec à Trois‐Rivières Trois‐Rivières Quebec Canada; ^2^ Canadian Practitioners Network for the Prevention of Extremist Violence (CPN‐PREV) Université du Québec à Montreal Montreal Quebec Canada; ^3^ Centre for the Study of Learning and Performance (CSLP) Concordia University Montreal Quebec Canada

**Keywords:** exposure to hate, hate speech, impact assessment, meta‐analysis, systematic review

## Abstract

**The Problem:**

People use social media platforms to chat, search, and share information, express their opinions, and connect with others. But these platforms also facilitate the posting of divisive, harmful, and hateful messages, targeting groups and individuals, based on their race, religion, gender, sexual orientation, or political views. Hate content is not only a problem on the Internet, but also on traditional media, especially in places where the Internet is not widely available or in rural areas. Despite growing awareness of the harms that exposure to hate can cause, especially to victims, there is no clear consensus in the literature on what specific impacts this exposure, as bystanders, produces on individuals, groups, and the population at large. Most of the existing research has focused on analyzing the content and the extent of the problem. More research in this area is needed to develop better intervention programs that are adapted to the current reality of hate.

**Objective:**

The objective of this review is to synthesize the empirical evidence on how media exposure to hate affects or is associated with various outcomes for individuals and groups.

**Search Methods:**

Searches covered the period up to December 2021 to assess the impact of exposure to hate. The searches were performed using search terms across 20 databases, 51 related websites, the Google search engine, as well as other systematic reviews and related papers.

**Selection Criteria:**

This review included any correlational, experimental, and quasi‐experimental study that establishes an impact relationship and/or association between exposure to hate in online and traditional media and the resulting consequences on individuals or groups.

**Data Collection and Analysis:**

Fifty‐five studies analyzing 101 effect sizes, classified into 43 different outcomes, were identified after the screening process. Initially, effect sizes were calculated based on the type of design and the statistics used in the studies, and then transformed into standardized mean differences. Each outcome was classified following an exhaustive review of the operational constructs present in the studies. These outcomes were grouped into five major dimensions: attitudinal changes, intergroup dynamics, interpersonal behaviors, political beliefs, and psychological effects. When two or more outcomes from the studies addressed the same construct, they were synthesized together. A separate meta‐analysis was conducted for each identified outcome from different samples. Additionally, experimental and quasi‐experimental studies were synthesized separately from correlational studies. Twenty‐four meta‐analyses were performed using a random effects model, and meta‐regressions and moderator analyses were conducted to explore factors influencing effect size estimates.

**Results:**

The 55 studies included in this systematic review were published between 1996 and 2021, with most of them published since 2015. They include 25 correlational studies, and 22 randomized and 8 non‐randomized experimental studies. Most of these studies provide data extracted from individuals (e.g., self‐report); however, this review includes 6 studies that are based on quantitative analysis of comments or posts, or their relationship to specific geographic areas. Correlational studies encompass sample sizes ranging from 101 to 6829 participants, while experimental and quasi‐experimental studies involve participant numbers between 69 and 1112. In most cases, the exposure to hate content occurred online or within social media contexts (37 studies), while only 8 studies reported such exposure in traditional media platforms. In the remaining studies, the exposure to hate content was delivered through political propaganda, primarily associated with extreme right‐wing groups. No studies were removed from the systematic review due to quality assessment. In the experimental studies, participants demonstrated high adherence to the experimental conditions and thus contributed significantly to most of the results. The correlational and quasi‐experimental studies used consistent, valid, and reliable instruments to measure exposure and outcomes derived from well‐defined variables. As with the experimental studies, the results from the correlation and quasi‐experimental studies were complete. Meta‐analyses related to four dimensions were performed: Attitudinal changes, Intergroup dynamics, Interpersonal behaviors, and Psychological effects. We were unable to conduct a meta‐analysis for the “Political Beliefs” dimension due to an insufficient number of studies. In terms of attitude changes, exposure to hate leads to negative attitudes (*d*_Ex_ = 0.414; 95% confidence interval [CI] = 0.005, 0.824; *p* < 0.05; *n* = 8 and *d*_corr_ = 0.322; 95% CI = 0.14, 0.504; *p* < 0.01; *n* = 2) and negative stereotypes (*d*_Ex_ = 0.28; 95% CI = –0.018, 0.586; *p* < 0.10; *n* = 9) about individuals or groups with protected characteristics, while also hindering the promotion of positive attitudes toward them (*d*_exp_ = −0.227; 95% CI = −0.466, 0.011; *p* < 0.10; *n* = 3). However, it does not increase support for hate content or political violence. Concerning intergroup dynamics, exposure to hate reduces intergroup trust (*d*_exp_ = −0.308; 95% CI = –0.559, −0.058; *p* < 0.05; *n* = 2), especially between targeted groups and the general population, but has no significant impact on the perception of discrimination among minorities. In the context of Interpersonal behaviors, the meta‐analyses confirm a strong association between exposure to hate and victimization (*d*_corr_ = 0.721; 95% CI = 0.472, 0.97; *p* < 0.01; *n* = 3) and moderate effects on online hate speech perpetration (*d*_corr_ = 0.36; 95% CI = –0.028, 0.754; *p* < 0.10; *n* = 2) and offline violent behavior (*d*_corr_ = 0.47; 95%CI = 0.328, 0.612; *p* < 0.01; *n* = 2). Exposure to online hate also fuels more hate in online comments (*d* = 0.51; 95% CI = 0.034–0.984; *p* < 0.05; *n* = 2) but does not seem to affect hate crimes directly. However, there is no evidence that exposure to hate fosters resistance behaviors among individuals who are frequently subjected to it (e.g. the intention to counter‐argue factually). In terms of psychological consequences, this review demonstrates that exposure to hate content negatively affects individuals' psychological well‐being. Experimental studies indicate a large and significant effect size concerning the development of depressive symptoms due to exposure (*d*_exp_ = 1.105; 95% CI = 0.797, 1.423; *p* < 0.01; *n* = 2). Additionally, a small effect size is observed concerning the link between exposure and reduced life satisfaction(*d*_corr_ = −0.186; 95% CI = −0.279, −0.093; *p* < 0.01; *n* = 3), as well as increased social fear regarding the likelihood of a terrorist attack (*d*_corr_ = −0.206; 95% CI = 0.147, 0.264; *p* < 0.01 *n* = 5). Conversely, exposure to hate speech does not seem to generate or be linked to the development of negative emotions related to its content.

**Author's Conclusions:**

This systematic review confirms that exposure to hate in online and in traditional media has a significant negative impact on individuals and groups. It emphasizes the importance of taking these findings into account for policymaking, prevention, and intervention strategies. Hate speech spreads through biased commentary and perceptions, normalizing prejudice and causing harm. This not only leads to violence, victimization, and perpetration of hate speech but also contributes to a broader climate of hostility. Conversely, this research suggests that people exposed to this type of content do not show increased shock or revulsion toward it. This may explain why it is easily disseminated and often perceived as harmless, leading some to oppose its regulation. Focusing efforts solely on content control may then have a limited impact in driving substantial change. More research is needed to explore these variables, as well as the relationship between hate speech and political beliefs and the connection to violent extremism. Indeed, we know very little about how exposure to hate influences political and extremist views.

## 1 PLAIN LANGUAGE SUMMARY

### 1.1 Exposure to hate in the media negatively affects how we think, feel, and act, but it's unclear how it connects to extreme views

Exposure to hate in the media negatively affects how we think, feel, and act, but it's unclear how it connects to extreme views.

### 1.2 What is this review about?

This systematic review seeks to determine the consequences for individuals or groups when they are exposed directly or as bystanders to hate speech online (websites, social networks, etc.) or through traditional media (newspapers, television, radio, etc.).

In this review “hate speech” refers to any type of communication in speech, writing, behavior, or multimedia, that attacks or uses pejorative or discriminatory language regarding a person or a group based on their protected characteristics, in other words, their religion, race, ethnicity, nationality, color, descent, and gender. Importantly, hateful rhetoric does not target individuals themselves but rather expresses feelings of disdain toward a collective.

To achieve this objective, we have searched for studies through various sources that present evidence on the impact of exposure to hate on people, as well as on the consequences with which this exposure to hate is associated.

Using this information, we conducted several meta‐analyses. A meta‐analysis is a type of scientific study that systematically synthesizes and analyzes the results of multiple previous research studies on a specific topic. Instead of conducting a new study, a meta‐analysis compiles data and results from several existing studies and combines them statistically to obtain an overall conclusion.

### 1.3 What are the main findings of this review?

We identified 55 studies that addressed this issue and identified 43 different consequences or outcomes of exposure to hate, which we classified into five major categories: Attitudinal changes, Intergroup dynamics, Interpersonal behaviors, Psychological effects, and Political beliefs. Using this information, we were able to perform 24 meta‐analyses on the first four categories.

In terms of attitude changes, exposure to hate leads to negative attitudes toward individuals or groups with protected characteristics. There is some evidence to suggest that it also may lead to negative stereotypes about these individuals or groups, while potentially hindering the promotion of positive attitudes toward them. However, it does not increase support for hate content or political violence.

Exposure to hate reduces trust between targeted groups and the general population but does not significantly impact the perception of discrimination among minorities. It is also linked to online victimization, offline violent behavior, and a contagion effect in online comments. Additionally, there is an indication of an association with online hate speech perpetration. Despite these negative effects, exposure to hate does not seem to directly affect hate crimes in specific areas, nor does foster resistance behaviors among individuals who are frequently exposed to it (e.g., the intention to counter‐argue factually). Psychological well‐being is significantly impacted, as exposure to hate causes depressive symptoms, reduces life satisfaction, and is associated with an increased social fear related to the likelihood of a terrorist attack. Nonetheless, it does not generate or contribute to the development of negative emotions related to its content.

### 1.4 What do the findings of this review mean?

This review confirms that when people are exposed to hate in the media, this exposure has a strong negative impact on them as individuals and potentially on the groups to which they belong. These findings are important for creating policies and interventions to prevent and address this problem. Hate speech propagates through biased comments and perceptions, normalizing prejudice and causing harm to the groups targeted. It can also lead to violence, victimization, and the perpetuation of these discourses among other individuals. Surprisingly, this research found that people exposed to hate speech do not necessarily feel increased outrage or disgust toward the content itself. This may explain why hate speech spreads easily, as people may be more inclined to relay material if they are not offended by it. It may also explain why some individuals perceive hate speech as not harmful, and therefore see no need to regulate it. Thus, public policies and interventions that focus solely on the content of hate speech may not produce significant change.

### 1.5 How up‐to‐date is this review?

The review includes studies published up to December 2021.

## 2 BACKGROUND

### 2.1 The problem

With the boom of interactive social media, hostile and offensive hate content has increased exponentially around the world (Weber et al., 2020). In surveys of young people between the ages of 15 and 30, as many as 53% of American, 48% of Finnish, and 39% of British respondents report having been exposed to hateful online material. In some cases, this exposure is also accompanied by victimization. For example, in the United Kingdom, 10–20% also report being an actual target of abuse (Vidgen et al., 2019). In New Zealand, the same is true for 11% of the adult population, while in the United States, as many as 41% of adults recount experiences of being victimized (Waqas et al., 2019).

Technological advancements such as the use of social networking to chat, search and exchange knowledge, express thoughts, and engage with others have rendered social media a convenient and effective platform of interaction (Rabah, 2014). However, the accessibility of popular social network sites like Facebook and Twitter (Mossie & Wang, 2020), the elective anonymity of cyberspace, and the ease with which divisive opinions can be expressed online (Davidson et al., 2019) are at least partly responsible for the spike in online hate content. Additionally, some studies have emphasized the influence of events of great social impact on the increase of hate speech. There is some evidence, for example, that terrorist attacks are followed by an increase in hate speech on the internet in the places where these attacks have occurred (Castaño‐Pulgarín et al., 2021; Kaakinen et al., 2018; Olteanu et al., 2018). Other events, such as Brexit in the United Kingdom or the Peace Accords in Colombia, which have been extremely polarizing for these countries, have been accompanied by waves of hate speech, fostered through disinformation (Castaño‐Pulgarín et al., 2021).

There is an important debate about the influence that the exposure of violence in the media and digital media could have on individuals. The Anderson‐Ferguson debate is the clearest example of this. Anderson and Bushman (2002) argue, for example, that there is a clear and positive link between violence and media and aggression regardless of the methods used. In another meta‐analysis, Anderson et al. (2010) conclude that there is strong evidence for the effect of video games on aggressive behavior, aggressive cognitions, and affect, as well as on decreased empathy and pro‐social behaviors. Ferguson (2015, 2017) considers these data to be at least inconsistent and, through other meta‐analyses, concludes that the evidence is weak or non‐existent. The root of the matter, according to the latter, author is the publication bias and moral debate around violence, that is, the tendency to publish only positive results while results showing no effects are not considered by researchers or published by specialized journals.

Although the precise determinants of such content can vary, research suggests the offline implications of digital hate speech are significant, with detrimental effects at the individual, community, and society levels (Del Vigna et al., 2017). Bliuc et al. (2018) conducted a systematic review of online racism and were able to identify some effects that this racism can have on exposed individuals. According to the studies identified by this systematic review, online racism has effects on the well‐being of people targeted by these discourses, mainly in terms of mental health, and can undermine social cohesion by perpetuating racial stereotypes and increasing political polarization. Other studies point to a clear association between digital hate speech and actual hate crime (Mossie & Wang, 2020), as well as online hate speech and offline violence against targeted communities (Weber et al., 2020). Moreover, according to a recent systematic review, exposure to radical violent online material is linked to extremist beliefs and possibly a higher risk of violent behaviors (Hassan et al., 2018).

In related fields, such as cyberbullying, the effects that direct victimization can have on exposed individuals, particularly in terms of mental health, have also been documented. In the same vein as the preceding studies, Fisher et al.(2016) found that cybervictimization among adolescents was moderately associated with suicidal ideation, depression, anxiety, self‐esteem problems, and self‐harm, and to a lesser extent, with substance use and social problems. These findings have been confirmed by the study of John et al. (2018), who found that children and youth who have been victims of cyberbullying were 2.35 times more likely to engage in self‐harm, 2.10 times more likely to exhibit suicidal behavior, 2.57 times more likely to attempt suicide, and 2.15 times more likely to have suicidal thinking.

Acts motivated by hate can have a broader impact on communities through vicarious victimization. In this process, individuals who are not direct victims of these acts, but who identify with the victims or share their characteristics, may also suffer negative consequences (Wenger et al., 2022). For example, this vicarious victimization has been linked to depressive symptoms (Wenger et al., 2022), as well as feelings of loneliness and a decrease in life satisfaction (Stahel & Baier, 2023). Additionally, hate‐motivated acts can affect those who are neither direct nor vicarious victims. Keel et al. (2022) found that people who are aware of hate crimes in their neighborhoods are more likely to anticipate ethnic discrimination and hold negative attitudes toward ethnic immigrants.

Interest in the reaction of bystanders exposed to hate speech has also recently increased, especially concerning their ability to intervene when such situations occur (Obermaier et al., 2023; Zapata et al., 2024). Personal factors such as empathy, previous victimization, and feelings of responsibility, as well as contextual factors such as the severity of the situation, social norms, the relationship with the victim, and the number of spectators, influence the propensity of adult and adolescent bystanders to intervene when witnessing online hate speech (Domínguez‐Hernández et al., 2018; Rudnicki et al., 2023). However, bystanders who frequently encounter hate content tend to become desensitized and intervene less in these situations (Castellanos et al., 2023; Obermaier, 2022). According to Castellanos et al. (2023), this frequent exposure affects the affective component of empathy, diminishing their emotional response.

Although hate content has been mostly associated with the Internet, traditional media have also been associated with the transmission of hate content, particularly in regions where the Internet has had a lower penetration, as well as in rural sectors. In Africa, for example, hate content broadcast on radio has been linked to violence and destabilization (Pate & Ibrahim, 2020; Somerville, 2011). The most extreme case of this connection has probably been Rwanda, where several researchers have pointed out the influence that hate content broadcast on the radio had on the genocide in the country (Adelman & Suhrke, 2017; Straus, 2007). In Western countries, hate content has been identified on local radio stations. This is the case of hate speech on commercial radio in the United States in which Latino communities are stigmatized and stereotyped (Noriega & Iribarren, 2012) or shock radio in the province of Quebec in Canada, which have been the source of the transmission of Islamophobic rhetoric in the country (B. Perry, Mirrlees, et al., 2017; B. Perry, 2019). Even though newspapers have paid less attention to these issues, some research has emphasized their role in the production of this content. According to Merklejn and Wiślicki (2020), traditional newspapers in Japan, for example, have played an important role in the development of right‐wing hate content targeting the Korean community in the country.

Preventing and countering hate speech is a huge challenge that involves taking into account multiple dimensions and interrelated actors. To curb the phenomenon of hate that is publicly spread through different media, governments are adopting laws to restrict the circulation of hate speech and pressuring internet platforms and social media platforms to design strategies to reduce its propagation (Mossie & Wang, 2020; J. Perry, 2017; Ross et al., 2016). However, despite this growing interest, there is a lack of agreement on what combating hate entails (Brown & Sinclair, 2020). One way to better understand the phenomenon and inform the policies and programs implemented to address it is through empirical research. This can provide evidence on the causes, consequences, and most effective solutions to this problem, as well as guide the development of work plans that fit the current reality of this problem.

However, this field of study is recent, and there are still large areas where little is known about this subject. Empirical evaluations of the impact of hate on different audiences, including individuals and communities, are, for example, notably limited and often rely on qualitative or content analysis (Castaño‐Pulgarín et al., 2021). Most existing studies, by contrast, have focused on identifying and analyzing the prevalence of harmful content, providing an understanding of how hate manifests and spreads across different media (Castaño‐Pulgarín et al., 2021). Although these studies are essential for recognizing the extent of the problem and the patterns of hate speech, they leave a significant gap in understanding its real and tangible effects on people. This includes how exposure to hate can affect mental health, interpersonal relationships, and social cohesion. Hence, a deeper knowledge of the impact of hate speech is crucial for addressing more complex issues and developing more effective intervention programs that are adapted to the current reality of hate.

Therefore, the proposed review aims to gather, analyze, critically appraise, and synthesize empirical research about the impacts or associations of exposure to, hate online and traditional media, specifically on individuals and communities. Results will inform policymakers and professionals working in this field about preventive measures and countermeasures to deal with the phenomenon, identifying gaps in the literature and helping to determine future research needs.

### 2.2 Defining hate speech and exposure to hate

A clear and comprehensive definition of hate speech is necessary to better understand this phenomenon. This definition serves as a guide for this review and makes it easier for the reader to receive and interpret the results appropriately. While there is no internationally reached consensus on what hate speech is, different entities have devised their own definitions. For example, the monitoring body of the European Commission against Racism and Intolerance (ECRI)—which has published individual country and cross‐country recommendations about the phenomenon's complex nature—states that hate speech entails:

the use of one or more particular forms of expression—namely, the advocacy, promotion or incitement of the denigration, hatred or vilification of a person or group of persons, as well any harassment, insult, negative stereotyping, stigmatization or threat of such person or persons and any justification of all these forms of expression—that is based on a non‐exhaustive list of characteristics or status that includes “race,” color, language, religion or belief, nationality or national or ethnic origin, as well as descent, age, disability, sex, gender, gender identity and sexual orientation. (2016, p. 16).

Meanwhile, the International Convention on the Elimination of All Forms of Racial Discrimination (UN Committee on the Elimination of Racial Discrimination CERD, 2013) understands hate speech as an utterance in direct disregard of human dignity and the core principles of human rights which seek to undermine both individuals and societies. For the United Nations, however, hate speech can be:

any kind of communication in speech, writing or behavior, that attacks or uses pejorative or discriminatory language with reference to a person or a group on the basis of who they are, in other words, based on their religion, ethnicity, nationality, race, color, descent, gender or other identity factor (2019, p. 10).

Also important to our understanding of hate speech are the voices of experts in the field and academics who highlight the difficulty of defining the phenomenon and identifying the conditions that allow its existence. Among these, Parekh's theorization (2012) stands out as the most relevant. According to him, hate speech is a form of communication

directed against a specified or easily identifiable individual or…a group of individuals based on an arbitrary and normatively irrelevant feature,” which “stigmatizes the target group by implicitly or explicitly ascribing to it qualities widely regarded as highly undesirable,” and portraying it as “an undesirable presence and a legitimate object of hostility. (pp. 40–41).

Against the numerous interpretations of hate speech, our definition draws from the UN's conception but lies in most agreement with Parekh's words. Referring to the UN definition ensures this review's alignment with the global consensus on hate speech. It also helps avoid confusion with other phenomena (e.g., legal hate speech vs. illegal hate speech). Nevertheless, recognizing the multifaceted nature and myriad manifestations of hate speech is crucial for this review as it greatly affects the inclusion/exclusion rationale and process.

The following definitions guide our systematic review:

**Hate:** “Hate” is defined by the urge to damage, humiliate, or destroy a targeted group or individual (White, 1996). Expressing hate toward the other has the objective not only to harm or be aggressive toward them but to eventually destroy the other either psychologically (e.g., via humiliation) or by literally getting rid of them (e.g., via killing and torturing) with the ultimate intention of damaging the target because of what they represent and not what or how they behave (Ben‐Ze'ev, 2008; Fischer et al., 2018).

**Hate Speech:** While hate speech is addressed in many international and regional standard‐setting documents, no internationally agreed‐upon definition of hate speech currently exists. “Hate speech” in this review refers to any type of communication in speech, writing, behavior, or multimedia, that attacks or uses pejorative or discriminatory language with reference to a person or a group based on their protected characteristics, in other words, their religion, race, ethnicity, nationality, color, descent, and gender. Importantly, hateful rhetoric does not target individuals themselves—as may be the case with cyberbullying—but rather expresses feelings of disdain toward a collective, even if this speech is directly targeted against an individual (Blazak, 2009; Hawdon et al., 2017).

**Interactions with Hate Speech:** In this review, “interactions with hate speech” encompasses three descriptors: “exposure to,” “active search for,” and “participation” in hate speech forums. Differentiating between these is important because some authors believe the effect differs depending on the type of interaction. For example, according to Schils and Pauwels (2014), active search leads to different outcomes than passive exposure. Therefore, for this review, “exposure to hate speech” relates to any encounter with material or messages, regardless of the medium, that promotes or incites hatred toward certain groups or individuals with protected characteristics. “Passive exposure” involves encountering hate content involuntarily through various forms of media. “Active exposure” or “Active search,” where individuals actively seek out hate content in the media. Lastly, we have considered “Participation,” which occurs when individuals engage in internet forums where such content is presented, even if they did not actively search for it themselves. Direct victimization in relation to this content was not considered as exposure in this study. Thus, we did not include studies that specifically focused on direct hate speech victimization.

**Hate via Media:** For the purposes of this review, “hate via media” means hate or hateful content accessible by any medium of communication designed to reach the general public. Moreover, the definition of “media” comprises traditional media (i.e., newspapers, radio, or television) and online media. The latter covers media appearing in either Web 1.0 (i.e., static HTML websites with minimal opportunities for users to interact or contribute content) or Web 2.0 (i.e., participative websites where users can interact with each other and contribute user‐generated content). Furthermore, Web 2.0 includes the following types of sites or apps: social networking sites with a social, professional, business, or ideological orientation; video‐ or image‐sharing sites; online discussion forums; wikis; blogs; multimedia messaging apps; search‐and‐discovery apps; any hybrids of the preceding; and sites within the deep web and dark web.

### 2.3 How exposure to hate may be linked to the outcomes

**Figure 1:**
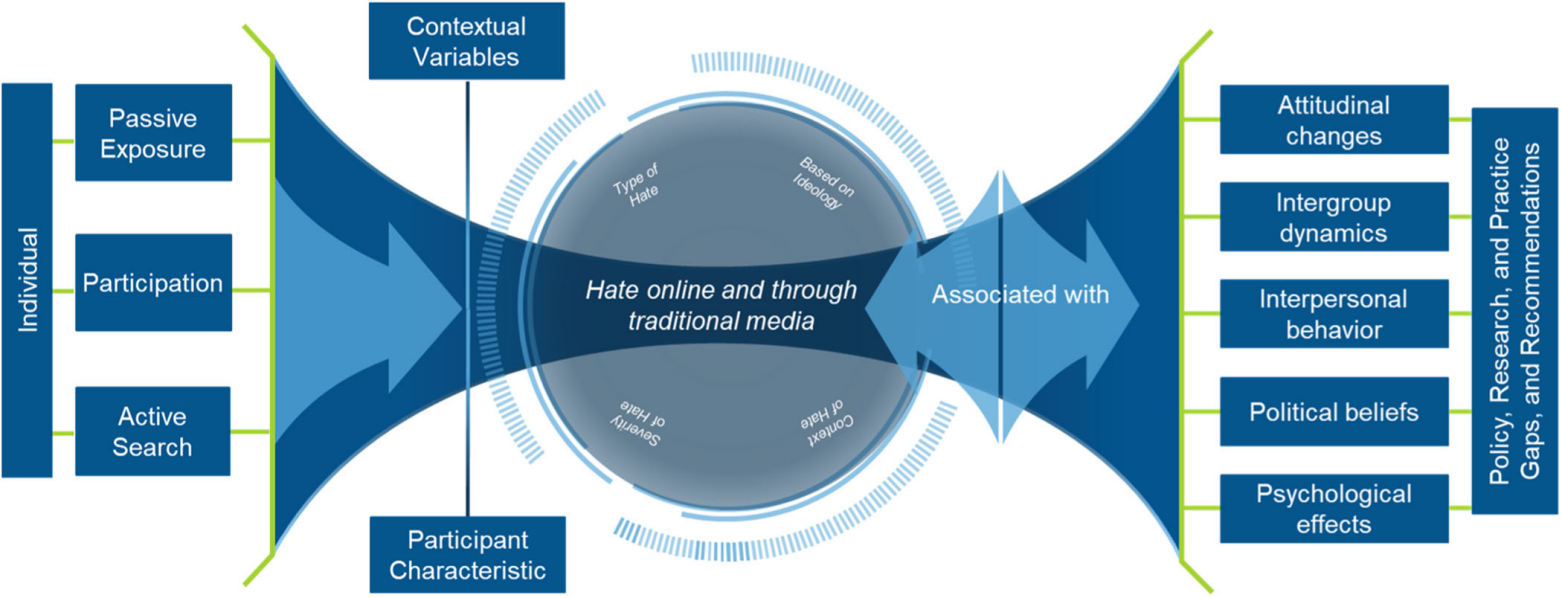
Logic model on impact of or association with hate online.

Hate content is omnipresent in both traditional media and online platforms, generating growing concern among academics, policymakers, and practitioners (Hawdon et al., 2017; Merklejn & Wiślicki, 2020; B. Perry, 2019; Straus, 2007; Tynes et al., 2008). To better understand how the exposure interacts with individuals through the media and thus better understand how the possible effects of this exposure can take place, we have developed a logic model that can capture the functioning of hate rhetoric and its consequences (see Figure 1). To develop the logic model, we relied on a combination of three conceptual frameworks: primarily cultivation theory (Busselle & Van den Bulck, 2017; Gerbner et al., 1994, 2002; Shrum, 1996) and social cognitive learning models (Bandura, 1986, 1989) to explain negative outcomes on individuals' cognitions and perpetration behaviors. Stress coping theory and trauma models were also used to explain the impact of exposure to trauma on negative emotional outcomes for victims.

According to the logic model, individuals may encounter hate online and in traditional media in a passive manner, such as when they come across hate content without actively seeking it. Alternatively, they may actively seek it out when intentionally searching for hate content. Their exposure can also be participatory, involving active engagement in spaces where hate content is propagated. Cultivation theory posits that such exposure, when repetitive, prolonged, or appealing, shapes an individual's perceptions, attitudes, and beliefs about others and society (Busselle & Van den Bulck, 2017; Gerbner et al., 1994; Shrum, 1996). Exposure to or consumption of hate speech can, for example, lead to changes in attitudes, particularly in relation to perceptions of outgroups and intergroup relations, perpetuating stereotypes and normalizing negative attitudes (Bliuc et al., 2018; Castaño‐Pulgarín et al., 2021). In online spaces, anonymity facilitates the expression of hateful sentiments with greater intensity than in the real world (Cohen‐Almagor, 2017; Gagliardone et al., 2016; Vollhardt et al., 2006). Exposure to hate is also associated with changes in political beliefs. Such exposure may influence individuals' willingness to vote for right‐wing political parties (Schmuck & Matthes, 2019) as well as increase agreement with extreme ideological statements (Wojcieszak, 2010).

Both cultivation theory and social cognitive learning models can be used to explain how, at a community level, exposure to online hate can erode social cohesion and diminish trust as well as fuel discrimination and intergroup conflict (Mosharafa, 2015; Petty & Cacioppo, 2015; Shrum, 1996; Wachs et al., 2022). Through social cognitive learning processes such as identification, imitation, and modeling, exposure to and consumption of hate can, in turn, lead to a higher risk of perpetrating hate and violence against targeted communities. This is because hate speech allows individuals and groups to express negative views toward others and coordinate efforts to act them out (Siegel, 2020) and because it can influence motivations to take action (Moule et al., 2017). One of the consequences of the COVID pandemic has been for example the proliferation of hate speech against Asian people, who in turn have suffered numerous assaults motivated by prejudice and negative attitudes (Gover et al., 2020). Some studies suggest that hate speech can have an important influence on the spread of new hate speech on the internet through comments (Spörlein & Schlueter, 2021), as well as hate crimes in the physical world (Nguyen et al., 2021). In some circumstances, hate speech can lead to extreme violence on a societal level. Hate speeches broadcast through the radio station RTLM in Rwanda have been identified, as previously mentioned, as one of the factors for the genocide in the country (Adelman & Suhrke, 2017; Straus, 2007). Violent radicalization has also been associated with consumption or exposure to this type of content. According to an African study, 42.5% of participants believe that hate messages could lead people to extremist positions and 27.5% to radicalization (Fayoyin, 2019). Thus, the data seem to indicate that traditional and social media could act as a breeding ground for real‐world violence, often directed against vulnerable minorities (Siegel, 2020).

Well‐established theoretical models such as stress and coping models (Lazarus & Folkman, 1984) and political violence trauma models (Herman, 2015) can help explain the negative psychological impacts of exposure to hate on victims. Specifically, exposure to hate may induce stress and emotional trauma, which may challenge a person or a community's coping strategies. Studies do report anxiety and depression to be associated with exposure to online hate material (Castaño‐Pulgarín et al., 2021). Specifically, exposure to online hate speech is linked to depression among African American youth (Tynes et al., 2008) and experiences of victimization are associated with self‐mutilation, suicidal ideation and behavior (John et al., 2018). In addition, some research claims that Muslims living in non‐Muslim Western societies are prone to anxiety as they anticipate online threats against them becoming a reality (Awan & Zempi, 2015). Over time, the individuals and the communities they identify with may become associated with the above‐mentioned outcomes, potentially affecting how societies function. Hate speech is known to compromise society‐level intergroup cohesion (Castaño‐Pulgarín et al., 2021) and result in mounting feelings of hostility and unease in intergroup contexts. (Adelman & Suhrke, 2017; Straus, 2007).

The literature thus provides support for our logic model demonstrating that exposure to hate speech can have multiple consequences for individuals and groups (Blaya, 2019; Bliuc et al., 2018; Castaño‐Pulgarín et al., 2021; John et al., 2018), particularly associated, as we will see in the findings section, with changes in attitudes, intergroup dynamics, behaviors, political beliefs, as well as being associated with mainly negative psychological effects. From this point of view, hate speech is not only a set of anodyne expressions but also actions that lead to concrete and harmful results in the real world (Salminen et al., 2020).

Surely the relation between exposure to hate online and in traditional media is a complex one, and impacts are likely mediated or moderated by characteristics of hate, including the type of content, ideologies, severity, frequency, and platform type. Costello and Hawdon (Costello & Hawdon, 2018) report a correlation between users' presence on Reddit and Tumblr and the frequency with which they may produce hateful online content. The latter is also linked to whether the users belong to an online community, particularly one already linked to hate speech. Furthermore, individual and contextual variables (e.g., individual traits, age, gender, socioeconomic background) can act as mediating factors when encounters with online hate are concerned. (Hawdon et al., 2017), for example, cite studies in which (1) individuals who are less guarded and easily trust others are more prone to experiencing online hate than individuals with other behavioral patterns and (2) women appear to be victimized online more often than men. Unfortunately, evidence on the prevalence of hate‐speech‐related abuse is not always readily available (e.g., due to elective anonymity on social media or individual and group characteristics of the perpetrators being largely unknown). Moreover, it often lacks the important contextual information necessary to better understand the phenomenon of hate speech (Vidgen et al., 2019).

### 2.4 Why it is important to do the review

The phenomenon of hate speech, its relationship with the media, and its ability to negatively affect offline contexts have been the subject of a wide range of studies, including some systematic reviews that have at least partially addressed these issues. Although there are both experimental and correlational quantitative studies, the existing literature has placed greater emphasis on qualitative and content analysis. Thus, to date, there have been no efforts to systematically collate, analyze and synthesize existing quantitative evidence on these impacts and associations. This is true for studies that focus on the dissemination of this content online, but it is even more true for studies that address its dissemination through traditional media, where there is no specific review on the topic.

This type of analysis has become particularly relevant on account of several government initiatives to restrain media platforms considered a breeding ground for violent extremism and other forms of violence (Gagliardone et al., 2016; Hussain & Saltman, 2014). In Europe, for example, the European Commission reached agreements in 2016 with the largest IT companies regarding a code of conduct obliging operators to promptly remove hate content online. Social media giants such as Facebook, Twitter, and Google are also being pushed to introduce some form of effective content control (Poletti & Michieli, 2018). In a similar vein, France's “Avia law” requires social media companies to promptly remove hate speech or else face fines. Meanwhile, New Zealand's “Christchurch Call” promises further development of tools that prevent users from uploading hateful content and greater transparency about how such content is being monitored and eliminated (New Zealand Government, 2019). Within the Canadian context, the government is also clarifying the definition of hate crimes, increasing funds for training related to online hate, and engaging online platforms and service providers to better monitor and address online hate speech and promptly remove illegal or criminal content (Standing Committee on Justice and Human Rights, 2019). The Canadian government has recently proposed a new law aimed at holding social media platforms accountable for harmful content on their sites, especially content affecting minors (Canadian Heritage, 2024).

Understanding the issue and impacts of hate speech is also of utmost importance because of their relationship to freedom of speech. If free speech is to be protected, it is crucial to build a deep and accurate appreciation of the links between hate speech and the actual harm it inflicts, an approach that will help present persuasive arguments in favor of new preventive measures (Barendt, 2019). For example, Canada's Digital Charter (2019) pledges to defend the freedom of expression while holding social media platforms accountable for hate speech online at the risk of incurring penalties (Government of Canada, 2019). The United Nations' Strategy and Plan of Action on Hate Speech (2019) urges nations to provide their digital citizens with the tools to identify, reject, and defend themselves against hate speech. At the same time, it emphasizes the right to freedom of expression and the importance of education when combatting hate speech online.

In this context, a comprehensive and systematic review of the impacts and associations of these discourses could be of great value. Indeed, the undertaking could provide a fulcrum on which governments, policymakers, and tech companies might base their efforts. As such, it could help them draw policies, reframe laws, and provide tools to prevent the negative consequences of hate speech.

To properly contextualize the upcoming work, we searched for prior relevant systematic reviews and meta‐analyses in ERIC, Academic Search Complete, and Google Scholar, using variations of search terms and concepts relevant to our study. Similarly, we searched for existing published reviews on the subject in the Campbell Library, the Cochrane Library, and the PROSPERO registry. Our searches yielded several studies relevant to our proposed review. However, we were unable to identify any other systematic reviews and meta‐analyses that synthesized studies posing similar research questions, despite the growing number of recently published systematic reviews. As we will see, all of these systematic reviews are limited to a broad narrative synthesis of this phenomenon.

Although our research team found reviews investigating specific outcomes of hate speech among specific populations and contexts, their understandings of hate speech were narrower than ours. For example, Bliuc et al. (2018) examined 10 years of research—conducted between 2005 and 2015—on cyber‐racism as perpetrated by groups and individuals, synthesizing a broad range of findings, methodologies, and key research areas. Our proposed review differs from Bliuc et al. (2018) in several ways. First, ours measures the effects (outcomes or associations) of these interactions on perpetrators *and* victims. Also, it is not limited to racism as a form of hate speech but includes all forms of hate speech targeted toward other protected characteristics such as gender, religion, or nationality, via both traditional and online media.

Likewise, Hassan et al. (2018) synthesized specific empirical research about the relation and effects of exposure to radical violent online material on related associations with extremist online and offline attitudes, emotions, and the risk of committing political violence. However, our proposed review includes studies on terrorist extremist speech and consumption and interaction with violent extremist content *only* when accompanied by hate speech and harmful communication toward protected characteristics and across different media types (i.e., online and traditional media channels).

Furthermore, Samari et al. (2018) systematically reviewed empirical research on Islamophobia as a form of discrimination and the resulting associations between Islamophobia, health, and socioecological determinants of health. However, as mentioned above, our proposed review is broader in terms of targeted groups. Also, while our review is limited to hate speech delivered via media, the resulting associations do not have been limited to health and socioecological determinants of health. Instead, we include all outcomes reporting participants' resulting behaviors, attitudes, and emotions.

In conducting our research, we were able to find reviews whose understanding of hate speech was similar to ours but whose overall aims, inclusion criteria, and linguistic reach differed significantly. Waqas et al. (2019), for example, conducted a mapping and scientometric analysis of research trends and hotspots in online hate research in studies published up to March 2019. These authors identified four main focus areas: cyberbullying, social media platforms, co‐morbid mental disorders, and profiling of aggressors and victims. They also observed a significant increase in the number of publications on this topic starting from 2005, with most of these publications originating from high‐income and Western countries. The findings of Waqas et al. provide a comprehensive overview of the prevalent research themes in this field. However, they do not address the research question our systematic review aims to answer.

Another bibliometric study is that of Tontodimamma et al. (2021), who carried out a broad mapping study of the conceptual structure of hate speech literature and the interactions of evolving themes over the last 30 years. To achieve these objectives, the researchers applied bibliometric measures, tools for mapping knowledge, and “mining” techniques to identify themes via recurring patterns of words. While the authors' definition of hate speech is close to ours, their main aim was to create a bibliometric overview of the breadth and limitations of current research related to hate speech. Such themes are directly pertinent to our review. However, we aim to offer a critical and in‐depth appraisal of the impacts and associations that interactions with hate—both online and through traditional media—can have on individuals and societies. Notwithstanding, Tontodimamma et al.'s study represents an important installment to the body of literature on the issue of hate speech. It also provides valuable insights from which our proposed review may benefit.

In another recent systematic review, Izquierdo Montero et al. (2022), examine the literature on this subject over the last two decades addressing similar topics to those of Waqas et al. (2019). This systematic review did not attempt to analyze the empirical results of studies on the subject, but rather to establish quite broadly which disciplines were involved in this study, which methodologies have been used, and which topics associated with hatred have been most addressed in the literature. Although it offers a general overview of the studies on the subject, the objectives of this study are completely different from those we set out to achieve with our systematic review and meta‐analysis.

Castaño‐Pulgarín et al. (2021) also conducted a large systematic review covering the period between 2015 and 2019 in which they sought to examine existing research on the role of the Internet and social media as potential facilitators of online hate speech. This systematic review has several differences from the systematic review we propose in this study. First, our approach focuses exclusively on the impacts or associations of hate exposure, whereas the Castaño‐Pulgarín et al. (2021) review covers the topic of the relationship between media and hate content more broadly, including quantitative and qualitative studies. Thus, its nature was more exploratory and more akin to what is known as a scoping review, which aims to identify the scope and extent of existing research on a topic. Second, our systematic review has a broader temporal range, as it incorporates studies up to 2021 with no initial limit, whereas the study by Castaño‐Pulgarín et al. (2021) is limited to studies published between 2015 and 2019. Third, due to the greater homogeneity of the topics and methodological designs included, Castaño‐Pulgarín et al. (2021) did not perform a meta‐analysis of the information collected, which is one of the objectives of our study.

The systematic review by Paz et al. (2020) offers a critical look at the evolution and current state of English and Spanish hate speech scholarship within the fields of communication studies and legal sciences. The review analyzes the studies according to impact factor, field of study, and language. However, it focuses primarily on the studies' objectives and methodologies. The authors stress that to be comprehensive and successful, approaches to countering the effects of hate speech need to include interdisciplinary and transversal collaborations. While the review represents a starting point for mapping hate speech scholarship in different countries and within varying disciplinary, thematic, and methodological fields, its reach is significantly narrower than that of our proposed review. Thanks to the multilingual nature of our research team, we include studies published in French, English, Arabic, Spanish, and Russian—giving our review a broader linguistic *and* cultural perspective—and do not impose a publication date limit. In addition, while we exclude other reviews, we broaden our reach by including mass‐media sources. More importantly, our review goes beyond aggregation and comparison, examining instead a wide range of *measurable* outcomes that encounters with hate can yield in an individual or group.

Kansok‐Dusche et al. (2023), in a recent systematic review, attempted to establish the prevalence of hate speech among adolescents and children and also to identify the definitions and constructs underlying prevalence assessments, as well as the theoretical and empirical relationship of hate speech to related concepts. Unlike our systematic review, this study focuses on the theoretical and conceptual aspects underpinning the definitions of hate speech used to estimate its prevalence among children and adolescents, rather than on the empirical findings of such research, which are the purpose of our study. Thus, this paper is theoretical and conceptual rather than empirical.

Finally and in the same vein as Kansok‐Dusche et al. (2023), the review by Poletto et al. (2021) examines how hate speech text is analyzed in the literature. The authors categorize studies on dimensions such as where the data was obtained, what type of behavior was represented, and what the annotation framework comprised. One of their key findings was a lack of consistency in hate speech datasets and taxonomies for harmful content aggregation. Given the field's heterogeneity, Poletto et al.'s review provides the research community with an up‐to‐date, multilingual, and multidimensional hate speech recognition resource. Our proposed review shares Poletto et al.'s concern over the need to support the research community by delivering a comprehensive, unbiased, and broad‐reaching review. However, its fundamental goal in our case is to produce a synthesis of empirical evidence on the effects and impacts of hate speech on individuals and societies.

In summary, our review aims to give practitioners and researchers a better understanding of how hateful rhetoric impacts targeted communities. It also provides evidence that may help those tasked with creating preventive measures to efficiently counter hate in an integrated manner at a time when both independently and in collaboration, nations are making concerted efforts to contain the phenomenon of hate speech. Furthermore, the review informs policymakers and professionals working in the field about existing strategic countermeasures to deal with the phenomenon, identify gaps in the literature, and help determine future research needs.

## 3 OBJECTIVES

The current review gathers, critically appraises and synthesizes empirical research about the impacts or associations of exposure to, consumption of, active search, or promotion of hate speech via media, at different levels and dimensions.

The general objectives of this review are as follows:

1. to critically and systematically synthesize the empirical evidence on the effects or impacts of exposure to or consumption, active search, or promotion of hate content online or in traditional media;
2. to collect and produce a meaningful classification of outcomes;
3. to describe how the characteristics of hate (e.g., type of content, ideologies, severity, type of platform) impact the documented effects;
4. to collect and identify the role of contextual variables (e.g., individual traits, age, gender, socioeconomic background) on the documented effects; and
5. to identify gaps and limitations in the research and related policy documents.

## 4 METHODS

The method used in this systematic review is mainly based on the protocol of Hassan et al. (2022). While we have adhered closely to the prescribed steps of this protocol, minor adjustments have been introduced. These will be described in a specific subsection at the end of this section.

### 4.1 Criteria for considering studies for this review

#### 4.1.1 Types of studies

We include any empirical study that is published up to December 2021 and employs primary data and quantitative measures to establish an impact and/or association relationship between exposure to or consumption of hate content on different platforms (online and traditional media) and the resulting consequences on individuals, communities, or society. To be eligible for inclusion studies have had to analyze instances of hateful communication that stigmatize individuals or groups based on their protected characteristics, such as religion, ethnicity, nationality, race, color, descent, or gender. As a result, this research excludes qualitative studies or sections of studies utilizing qualitative methods, as well as research exclusively relying on basic descriptive statistics for single variables, content analysis, opinion‐based or theoretical studies, or studies lacking primary data.

This research encompasses a diverse range of study designs, including experimental or quasi‐experimental designs, as well as cross‐sectional and longitudinal studies with or without control groups. Specifically, we have included:

- Experimental Designs: We included between‐subjects randomized controlled trials with at least one condition in which the treatment group was exposed to hate content.
- Quasi‐Experimental Studies: We include quasi‐experimental studies with two or more comparative groups wherein these groups are either exposed to or consume hate content, alongside control groups. These studies compare at least one outcome between the groups and include retrospective case‐control studies, comparative studies employing a post‐test‐only design, and studies comparing a group before and after exposure to or consumption of hate content.
- Cross‐sectional and Longitudinal Correlational Studies: These studies employ appropriate statistical methodologies such as multivariate regression and bivariate correlation models, providing adequate information to facilitate the calculation of effect sizes. This category includes studies based on a single sample, both cross‐sectional and longitudinal, where a discernible association is established between observed variations in exposure to or consumption of hate content and one or more outcomes of interest.

Given the diverse types of study designs included in our research—experimental, quasi‐experimental, and correlational—our approach acknowledges the complexity of analyzing the impact of hate content across different platforms and contexts. The inclusion of multiple study designs allows us to capture both controlled, experimental insights and more naturalistic, observational data, which is crucial given the nature of hate content as a public phenomenon. The relationship between exposure to hate content and its impacts is not confined to the artificial settings typically found in experimental studies. Instead, it also involves real‐world contexts where individuals encounter hate content, either directly or as bystanders, across online and traditional media. To properly understand these dynamics, it is essential to consider studies conducted in natural settings, such as social media platforms, where hate content is propagated and experienced. This aligns with recommendations by Caudy et al. (2016), who suggest incorporating a variety of designs, including both experimental and observational, in meta‐analyses to ensure a more comprehensive understanding of the phenomenon.

To ensure a comprehensive evaluation, no further constraints have been imposed in terms of study design or publication date, given the need to encompass the entirety of existing literature in this field.

Regarding the sampling methods utilized in the primary studies incorporated within this systematic review, a wide range of approaches has been deemed acceptable, provided that the sampled population is adequately described, enabling meaningful inferences about the intended population of the study.

#### 4.1.2 Types of participants

To better understand the heterogeneous impact of hate content in the media at different levels and dimensions, we did not place limits on the population or individual characteristics of the study participants. However, in addition to studies based on individuals, this systematic review includes studies based on the analysis of comments or posts in social media and studies that compare the latter in relation to precise geographical areas such as cities or municipalities (ecological studies). That is, studies whose unit of analysis is not based on an individual's direct response to a data collection tool.

#### 4.1.3 Types of exposure to hate

The studies included in this review have examined hate speech delivered through any media designed to reach the general public. We have defined “exposure to hate content” as any encounter with material or messages, regardless of the medium, that promote or incite hatred toward certain groups or individuals with protected characteristics. In this systematic review, we have examined three distinct types of exposure. The first type is passive exposure, which involves encountering hate content involuntarily through various forms of media. The second type is active exposure, where individuals actively seek out hate content in the media. Lastly, we have considered participation, which occurs when individuals engage in internet forums where such content is presented, even if they did not actively search for it themselves. Direct victimization in relation to this content was not considered as exposure in this study. Thus, we did not include studies that specifically focused on direct hate speech victimization.

We have included two types of media: traditional mass media (i.e., newspapers, radio or television) and online media. The latter covers media appearing in either Web 1.0 (i.e., static HTML websites with minimal opportunities for users to interact or contribute content) or Web 2.0 (i.e., participative websites where users can interact with each other and contribute user‐generated content). In addition to these two types of media, we have included studies that have analyzed any type of political propaganda broadcast through any media that can be considered as containing hate speech.

#### 4.1.4 Types of outcome measures

The outcomes of interest in this review are the measurable effects of individuals' interactions with hate speech through the media. These effects can be measured through self‐reported measures (i.e., when individuals interviewed report their own outcomes or perceptions of outcomes) and measures reported by peers, family members, or professionals, along with measures reported by governments, law enforcement agencies, and data generated from open sources. This includes data that has been obtained through social media, such as Twitter, Facebook, YouTube, etc.

These results of interest can be classified into five broad categories or dimensions:

- Attitudinal change: This category refers to changes in the attitudes, beliefs, or opinions of individuals toward a particular content or group concerning or caused by exposure to hate.
- Intergroup dynamic: This term includes a wide array of processes and relationships that shape interactions and perceptions between social groups, alongside the determinants that influence trust and attitudes toward diverse groups within a community, especially in the context of groups targeted by hate speech and the majority groups.
- Interpersonal behavior: This dimension refers to behavioral changes in individuals and groups in relation to or caused by exposure to hate.
- Political beliefs: This category refers to changes in individual or group political beliefs or ideas related to or caused by exposure to hate.
- Psychological effects: these outcomes refer to changes in individual or group emotions or symptoms related to or caused by exposure to hate.

In the published protocol of this systematic review (Hassan et al., 2022), we organized the potential outcomes based on the individual, community, and societal levels. Although this original classification is still relevant, we consider that the five dimensions proposed are more appropriate for this study. A large majority of these outcomes are totally or partly related to the individual level, particularly psychological effects, attitudinal changes, interpersonal behaviors, and political beliefs. However, it is much more difficult to determine outcomes at the community and societal level. Intergroup dynamics, for example, focus on impacts on intergroup relations that may in turn have consequences at the community and societal levels. On the other hand, studies that are not based on individuals may be the most concrete approximation of the impact at the societal level, since they analyze information at the most global level, that is, comments on social networks or hate crimes.

#### 4.1.5 Types of settings

The inclusion of studies has not been limited with respect to the settings.

#### 4.1.6 Language of studies

We have included any documents written in English, French, Arabic, Spanish, and Russian (languages spoken by the research team members).

#### 4.1.7 Exclusion criteria

The following types of studies have been excluded:

- Studies that do not analyze exposure to hate content through the media and its effects.
- Descriptive, opinion, and theoretical documents on the subject or studies that do not use primary data.
- Systematic reviews and literature reviews.
- Studies on programs that aim to prevent hate online or offline.
- Studies that analyze direct victimization through hate speech.
- Any qualitative study or any section of a mixed‐method study using qualitative methods.
- Studies based in content analysis.
- Studies based exclusively on basic descriptive statistics.

### 4.2 Search methods for identification of studies

#### 4.2.1 Electronic searches

To locate relevant research literature, we have employed a professional librarian to assist in developing an effective search. The searches targeted online hate, with groups of search terms formed around the concepts of (1) Hate, (2) Expression of Hate, (3) Hate Source, and (4) Media Environment. Where possible, proximity operators have been used to closely link the concepts of hate and the expression of hate. The complete search string terms can be found in Supporting Information S1: Appendix A.

Most search terms were searched in the main fields of each database (not full text), except for media‐related search terms, which were searched in the abstracts. In this case, we have searched for media only in the Abstract field. There are two reasons for this. First, the use of social media in research is increasing in several areas of knowledge, and the potential number of studies identified in the initial step of the search could make this systematic review impracticable due to the limited resources available to us. Second, the media environment is a key aspect of this research, and the studies to be included in this review should emphasize at the outset that they examine hate speech delivered through any media designed to reach the general public.

Next, we conducted searches in a variety of bibliographic databases, both subject‐specific databases and general multi‐disciplinary databases, in two periods. The first search was carried out on 27 May 2021, and the second between 19 and 20 September 2022. The proposed database list is as follows:

- Academic Search Complete (hosted on EBSCO).
- Communication Abstracts (hosted on EBSCO).
- Communication and Mass Media Complete (hosted on EBSCO).
- Criminal Justice Abstracts (hosted on EBSCO).
- Education Resources Information Center (ERIC, hosted on EBSCO).
- MEDLINE (hosted on OVID).
- National Criminal Justice Reference Service (NCJRS, hosted on ProQuest).
- Political Science Complete (hosted on EBSCO).
- ProQuest Central.
- ProQuest Dissertations and Theses Global.
- PsycINFO (hosted on APA PsycNet).
- Social Services Abstracts (hosted on ProQuest).
- Sociological Abstracts (hosted on ProQuest).
- SocINDEX (hosted on EBSCO).
- Web of Science Core Collection.
  ○ Science Citation Index Expanded (SCI‐EXPANDED).
  ○ Social Sciences Citation Index (SSCI).
  ○ Arts & Humanities Citation Index (A&HCI).
  ○ Conference Proceedings Citation Index—Science (CPCI‐S).
  ○ Conference Proceedings Citation Index—Social Science & Humanities (CPCI‐SSH).
  ○ Emerging Sources Citation Index (ESCI).

While the searches have employed standard Boolean logic, they have been tailored to the features of each database, making use of available controlled vocabulary and employing proximity operators where possible. The exact search string for each database is available in Supporting Information S1: Appendix D.

#### 4.2.2 Searching other resources

The searches of bibliographic databases had been supplemented with a targeted search for gray literature, utilizing the Google search engine as the primary tool. Additionally, the OpenGrey collection had been consulted, along with the websites of various governments and non‐governmental organizations (refer to Table 1). In the case of websites, research assistants manually searched for papers and research reports in the documentary sections of each organization's website. At this initial stage, any document related to research on hate was included in the initial screening.

**Table 1:** List of gray literature sources.

ADL (Anti‐Defamation League) (https://www.adl.org/) Alternatives to Violence Project (https://avp.international) Article 19 (https://www.article19.org/) BRICKS ‐ Building Respect on the Internet by Combating hate Speech (https://www.bricks-project.eu) CHRC (Canadian Human Rights Commission) (https://www.chrc-ccdp.gc.ca/eng/) Council on Foreign Relations (https://www.cfr.org/) Counter Narratives (http://www.counternarratives.org/) Dangerous Speech Project (https://dangerousspeech.org/) eMORE (Monitoring and Reporting Online Hate Speech in Europe) (https://web.archive.org/web/20190209185046/https://www.emoreproject.eu/) Equality and Human Rights Commission (https://www.equalityhumanrights.com/) German National Center for Crime Prevention (https://www.nzkrim.de/english) Global Center on Cooperative Security (https://www.globalcenter.org) Global Kids Online (http://globalkidsonline.net/) Hatebase (https://hatebase.org/) Hedayah (https://www.hedayahcenter.org/) HRMI (Human Rights Measurement Initiative) (http://hrmi.lt/en/) Human Rights Watch (https://www.hrw.org/) IFEX (International Freedom of Expression Exchange) (https://ifex.org/) ILGA‐Europe (The International Lesbian, Gay, Bisexual, Trans and Intersex Association) (https://www.ilga-europe.org/) INACH (International Network Against Cyber Hate) (https://www.inach.net/) INAR (Irish Network Against Racism) (https://inar.ie/) International Centre for Counter‐Terrorism—The Hague (ICCT) (https://icct.nl) International Network for Hate Studies (https://internationalhatestudies.com/) ISD (Institute for Strategic Dialogue) (https://www.isdglobal.org/) ISTSS (International Society for Traumatic Stress Studies) (https://www.istss.org/) KAICIID Dialogue Centre (https://www.kaiciid.org/) MANDOLA (Monitoring and Detecting Online Hate) (http://mandola-project.eu/) MediaSmarts (https://mediasmarts.ca/) Minority Rights Group International (https://minorityrights.org/) Moonshot (http://moonshotcve.com/) OHCHR (Office of the United Nations High Commissioner for Human Rights) (https://www.ohchr.org/EN/) Online Antisemitism Task Force (https://www.antisemitismtaskforce.org/) OPHI (Online Hate Prevention Institute) (https://ohpi.org.au/) OSCE (Organization for Security and Co‐operation in Europe) (https://www.osce.org/) Partners Against Hate (http://www.partnersagainsthate.org/) Report It (https://www.report-it.org.uk/) Research Outreach (https://researchoutreach.org/) Tech Transparency Project (https://www.techtransparencyproject.org/) The Alan Turing Institute (https://www.turing.ac.uk/) The Council of Europe (https://www.coe.int/en/) UiO C‐REX ‐ Center for Research on Extremism (https://www.sv.uio.no/c-rex/english/) UK Home Office Research Database (https://www.gov.uk/government/organisations/home-office/about/research) UK Safer Internet Centre (https://www.saferinternet.org.uk/) UNAOC (United Nations Alliance of Civilizations) (https://www.unaoc.org/) UNCCT (United Nations Counter‐Terrorism Centre) (https://www.un.org/counterterrorism/cct) UNESDOC (https://unesdoc.unesco.org/) UNICRI (United Nations Interregional Crime and Justice Research Institute) (http://www.unicri.it/) UNODC (https://www.unodc.org/) US National Criminal Justice Reference Service (https://www.ncjrs.gov) Project SOMEONE (https://projectsomeone.ca) No Hate Speech Movement (https://www.coe.int/en/web/no-hate-campaign/no-hate-speech-movement)

*Source*: Authors.

The websites and proceedings of any identified academic conferences considered relevant were also scanned. To ensure a comprehensive review, we additionally used backward citation searching, which involved examining the reference lists of included articles and prior reviews in the subject area (Bliuc et al., 2018; Carthy et al., 2020; Castaño‐Pulgarín et al., 2021; Hassan et al., 2018; Waqas et al., 2019; Windisch et al., 2022; Wolfowicz et al., 2022) to identify additional relevant studies. This approach allowed us to uncover important research that might have been missed in the initial search due to variations in search terms or database coverage. We also contacted experts in the field.

### 4.3 Data collection and analysis

#### 4.3.1 Selection of studies

The process of selecting admissible evidence studies was conducted by two research assistants (Ph.D. students in social sciences disciplines) who independently screened the abstracts of the total selected studies identified in the literature search. In this initial step, the research assistants applied four criteria to assess the eligibility of the studies:

1. Do the studies directly address hate speech as it has been defined in this protocol?
2. Do the studies measure exposure to or consumption of hate speech through a media source?
3. Do the studies use any empirical and primary data?
4. Do the studies analyze any impact or association of this hate content on individuals and communities?

All questions could be answered through one of three alternatives: “yes,” “no,” or “maybe.” If one of the criteria was not met (alternative “no”) for both reviewers, the study was excluded at this stage. In this first step, Cohen's *κ* was computed to ensure adequate inter‐rater agreement. The resulting coefficient was 0.581, which slightly fell below the minimum threshold recommended. Consequently, all discrepancies between the reviewers were further examined in a second screening phase. If the disagreement persisted, a final decision was made by the principal researchers. Thus, all studies that were at least classified as “maybe” on all these criteria were selected for full‐text screening. The research assistants also checked for duplicate sources.

During the full‐text screening, the assistants confirmed that the studies met the four initial criteria, which is often not easy at the initial stage, as well as the full eligibility criteria described earlier in the protocol. All studies categorized as “maybe” were transformed into “yes” or “no.” In addition, they also confirmed that the hate content addressed protected characteristics of individuals or groups, that the studies were based on eligible study designs, that the studies provided a bivariate or multivariate analysis of this association, and that it was not a duplicate source. If all of these were confirmed, the selected studies were coded in their entirety.

Lastly, the PRISMA (http://www.prisma-statement.org) template was used to record the results of the literature searches in a flowchart.

#### 4.3.2 Data extraction and management

A coding sheet (see Appendix B in the Supporting Information document) including the following criteria for data extraction was created for each study:

– Reference information: Document ID, Study Title, Study Author(s), Publication Year, Place published or accessed with URL, Reference Type, Coding References.
– Study details: Country of Study, Language, Date of Research, Peer reviewed, Funded research, Conflicts of interest, Ethical Issues.
– Methodology: Type of study, Type of design, Sampling procedure, Country/place of recruitment, Sample characteristics, Source of hate speech measure, Source of outcome measure, Quantitative measures on the link between exposure and outcome, Qualitative measures on the link between exposure and outcome.
– Independent Variable Details: Perpetrator of Hate Speech, Target of Hate Speech, Type of Hate Speech, Participants' interaction with hate speech (Exposure to hate, Active search of hate, Participation in forums), Hate Speech medium (Traditional media, Online media, Political propaganda).
– Dependent variable details: Participants' outcome after interaction with hate speech (Type of measured outcome: Attitudinal change, Intergroup dynamic, Interpersonal behavior, Political beliefs, or Psychological effects).
– Interaction variables measured: i.e. interaction, confounding, or moderating variables that influence the relationship between the independent and dependent variables. Quantitative results on the link between exposure and outcome: Statistical results such as effect size or any other statistic that allows us to calculate this effect size as well as unexpected outcomes including study harms.
– Qualitative results on the link between exposure and outcome: Relevant qualitative results that allow the analysis, interpretation, or contextualization of the results obtained by the studies. Authors' Conclusion and Recommendations (policy, research, practice).
– Study limitations and ethical issues.

#### 4.3.3 Assessment of risk of bias in included studies

Given that this systematic review includes both experimental and nonexperimental studies, we have chosen the Mixed Methods Appraisal Tool – MMAT (Hong et al., 2018). This tool was initially developed to evaluate mixed methods studies; however, it includes two sections to individually evaluate quantitative randomized controlled trials and quantitative non‐randomized studies, which are the two main design types included in this review. The MMAT includes a broad variety of studies under the label “Quantitative non‐randomized studies.” This kind of study is an intermediate category between randomized control experimental trials and basic quantitative‐descriptive studies. Therefore, this category encompasses all the correlational and quasi‐experimental studies that were selected for the review. This tool enables a comprehensive analysis of the issues (adequate randomization, baseline equivalence of groups, sample representativeness, adjustment for confounding factors, etc.) that may affect the selection of studies in this review.

Each section includes five criteria for each type of design. In the case of randomized controlled trials, we choose not to consider one of these criteria, which consists of determining whether the persons assessing the effects of the exposure are unaware that they have been exposed (Are outcome assessors blinded to the intervention provided?). This is hardly applicable to the type of study we are evaluating. Thus, in the case of these studies, we have only used four of the five recommended criteria.

Two trained reviewers evaluated the studies, and Cohen's *κ* was recalculated to measure the level of agreement between them. The resulting Cohen's *κ* value ranged between 0.62 and 0.69. In certain cases, it was not possible to calculate Cohen's *κ* due to one of the variables being a constant. Regardless of Cohen's *κ* results, any disagreements in the evaluation of the studies were resolved by one of the principal investigators. No studies were excluded based on methodological weaknesses during this phase.

The tool and the risk of bias assessment of the included studies are presented in a table showing the results per study (Yes, No, I can't tell) for each variable of the MMAT. These results are explained in detail in the corresponding results section. The table and the criteria of MMAT can be seen in Appendix C (see Supporting Information document).

#### 4.3.4 Exposure effect measurements and calculation of effect sizes

As already mentioned in the corresponding section, to calculate effect sizes, the relevant statistics, such as means, standard deviations, sample sizes in all conditions, etc., were extracted from the retained papers. In some cases, this information was also found in the published supplementary information documents or through the authors of the studies.

The effect sizes were first calculated in relation to the type of design and the statistic used in the studies and then transformed into the standardized mean difference also called Cohen's *d*. A reason for this choice is that the studies included in the metric employ different scales to report the outcomes (Borenstein et al., 2009). This metric therefore ensures comparability of results across studies.

For experimental studies (randomized and not randomized) from studies using two independent groups, we estimate the standardized mean difference as follows:

$$d=\frac{\underline{X_{1}}-\underline{X_{2}}}{S_{\mathrm{pooled}}},$$

where $\underline{X_{1}}$ is the mean of the treatment sample, $\underline{X_{2}}$ the mean of the control group and $S_{\mathrm{pooled}}$ the standard deviation within groups, pooled across groups and defined by:

$$S_{\mathrm{pooled}}=\sqrt{\frac{\left(n_{1}-1\right){S_{1}}^{2}+\left(n_{2}-1\right){S_{2}}^{2}}{n_{1}+n_{2}-2}},$$

where *n*_1_ and *n*_2_ are the sample sizes in the two groups, and *S*_1_ and *S*_2_ are the standard deviations in the two groups (Borenstein et al., 2009).

For correlational studies, all effect sizes were first calculated as the correlation coefficient *r*. We used bivariate correlations as our main method of estimating the effects of different variables, following the previous literature on similar topics (Wolfowicz et al., 2021), because this method is reliable and consistent across different studies. We obtained bivariate correlations mainly from zero‐order correlation matrices, but we also calculated them from summary statistics such as *t*‐tests, *F*‐tests, *χ* ^2^ tests, and ANOVAs, as well as other conventional hypothesis tests, when the matrices were not available. To calculate *r* from these statistics, we used the “Practical Meta‐Analysis Effect Size Calculator [Online calculator]” of the Campbell Collaboration website,^1^ which is based on the formulas provided by Lipsey & Wilson (2001).

We then converted the correlation coefficient *r* to Cohen's *d* using this formula.

$$d=\frac{2r}{\sqrt{1-r^{2}}}.$$

For studies in which we only had access to the coefficients of multivariate statistical models, partial effect sizes were estimated using the Online calculator, following the formulas below inspired by Borenstein et al. (2009) and Wolfowicz et al., (2020, 2021).

– In cases where the independent and dependent variables were dichotomous variables and only *β* was reported, Cohen's *d* was calculated as follows:
$$d=\beta \left(\frac{\sqrt{3}}{\pi }\right).$$
– For linear regression models where both independent and dependent variables are continuous, *r* was first calculated and then converted to *d* according to the previous formula. In this situation, *r* was calculated as follows:
$$r=\frac{{SD}_{X\;}\beta }{{SD}_{Y}}.$$
– In cases in which the standard deviation was not reported, particularly in situations where the independent variable was dichotomous and the dependent variable was continuous, as well as in situations in which the independent variable was ordinal or continuous and the dependent variable was dichotomous, we proceeded to calculate *r* using the ratio *t* = *β*/*SE* and the following formula

$$r=\frac{t}{\sqrt{t^{2}+n}}-2.$$

In all cases, the meta‐analyses were conducted only when the exposure was measured using the same type of variable.

We had initially considered synthesizing longitudinal studies independently. However, we identified only two noncomparable studies. These studies used a pre‐post model with one (Lee & Leets, 2002) or three follow‐ups (Schmuck & Tribastone, 2020). We then decided to include them in the meta‐analyses. For this purpose, we only consider as a comparable reference the results of the first post‐exposure measure, to make them comparable with other pre‐post studies included in this review.

We also included, as indicated in the “Types of Participants” section, studies that were not based on individuals. These studies examined the association between online expressions of hate or support or the association between these expressions of hate and the presence or absence of hate crimes in certain locations. These studies were synthesized independently. For retrospective case‐control studies, using logistic regressions, we calculated odds ratios (ORs) from the model estimation coefficients and then converted them to Cohen's *d*. However, when it was not possible to estimate the odds ratios from the model coefficients (because of the models used by the authors), we used the transformations presented above to calculate the effect sizes.

#### 4.3.5 Unit of analysis issues and independence of effects sizes

One of the main problems in a meta‐analysis is the potential dependence on effect sizes that may lead to a higher weighting of the final calculated effect size (Lipsey & Wilson, 2001). This dependence on effect sizes must therefore be reduced or eliminated. In the case of this systematic review, the basic unit of analysis was an effect size that accounted for a specific association or causal relationship between exposure to hate in a media outlet and a potential effect. Each study could potentially report more than one such relationship and thus there could be a risk of including two associations based on the same sample. To avoid this, we first listed all outcomes for each study, including an operational definition of each, and constructed specific categories of these that could be synthesized into a meta‐analysis (see the list of outcomes in Table 3). Second, the sample information from each study was carefully examined to avoid that two or more identical outcomes based on the same sample were synthesized in the same meta‐analysis. Thus, a single study analyzing the same association or causal relationship was included in each meta‐analysis. Whenever it was identified that more than one study or publication reported the same outcome derived from the same data set, the study published in a peer‐reviewed journal containing more detailed information was always retained for the meta‐analysis.

#### 4.3.6 Dealing with missing data

In cases in which the data necessary to conduct a meta‐analysis were not present in the studies, the following measures were taken:

– Search for supplementary material.
– Contact the author(s) and ask them to provide the missing data.

In 10 studies we obtained the necessary information directly from the authors. In the other cases, especially in the multivariate studies in which it was not possible to obtain information on the total effects, the analysis was performed with the partial effects, following the procedure described above.

#### 4.3.7 Assessment and investigation of heterogeneity

We used several methods to evaluate the heterogeneity of the data in each meta‐analysis. These included Cochran's *Q* (and its corresponding *χ* ^2^ value), *τ* ^2^, the *I* ^2^ statistic, and the prediction interval. A significant *Q* value suggests possible heterogeneity. On the other hand, an *I* ^2^ value of 0 indicates no heterogeneity. Following Borenstein (2019), we used the prediction interval to determine the degree of data dispersion within a meta‐analysis.

When we found significant heterogeneity and there were enough studies, we tried to identify its potential sources by conducting meta‐regressions for continuous variables and moderation analyses for categorical variables. We only performed these analyses when meta‐analyses had at least five studies. We used meta‐regression for two continuous variables: the proportion of male participants in the sample and the average age of the sample. We also wanted to include the year of data collection, but this information was not reported sufficiently in the selected studies.

We performed moderation analysis with three categorical variables: the type of media involved, the geographic region, and the classification of the exposed individuals. The media types examined in this review included traditional media, political propaganda, and Internet social media. Since most of the selected studies focused on Western countries, we made a distinction between those based on samples from the United States and those from other countries. Moreover, we categorized exposed individuals as belonging to a minority group frequently targeted by hate content or to a nonspecific group. The type of exposure was initially thought to be a potential moderator variable. Three different types of exposure were distinguished: passive exposure, active search of hate content, and involvement in online forums where such content was common (Participation). However, only two studies in this systematic review explicitly linked themselves to active search of hate content or involvement in online forums. Therefore, the type of exposure as a moderator variable became irrelevant.

#### 4.3.8 Assessment of reporting biases

The publication bias was assessed using the Trim and Fill method (Duval & Tweedie, 2000a, 2000b) and the Egger regression (Egger et al., 1997). In the case of the Trim and Fill method, asymmetric funnel plots are identified as indicators of publication bias. The method consists of three steps: trimming, filling, and recalculating. First, the method trims off the smallest studies on one side of the funnel plot that make it asymmetric. Second, the method fills in the missing studies on the other side of the funnel plot by imputing their effect sizes based on a chosen estimator. Third, the method recalculates a new overall effect size by including the filled studies. The method repeats these steps until the funnel plot becomes symmetric around the new effect size (Borenstein et al., 2009). In the case of Egger regression, when the intercept estimate is greater than 0 and significant, it is considered that there is a possible publication bias. Both analyses, however, depend on enough studies per meta‐analysis to be reliable.

#### 4.3.9 Data synthesis

The meta‐analysis was performed using Biostat Comprehensive Meta‐Analysis (CMA) version 4 software (Borenstein et al., 2009). Because the data from the studies included in this SR were from samples from different populations, random‐effect models were used to account for this heterogeneity.

As mentioned, we classified each outcome based on an exhaustive review of the operational constructs contained in the studies. A separate meta‐analysis was performed for each of the identified outcomes that originated from different samples. We present the results of the meta‐analyses as standardized difference‐in‐means with 95% confidence intervals in a series of ordered tables, with each row representing a separate analysis for each given outcome (see Tables 5, 6, 7, 8, 9).

The studies included in this systematic review analyzed information from three types of samples: individuals who have interacted with hate content through media, comments, or posts on social networks where this type of content existed, and the number of hate crimes in specific geographic areas (ecological studies). Each case was analyzed separately, despite the potential to evaluate the same outcome. This separation is due to the existence of three different types of samples, each operating at distinct scales. Consequently, the principle of independence of effect sizes is applied individually to each case.

Within the individual‐based studies, experimental and quasi‐experimental studies were synthesized together, since they had in common that exposure was controlled, and they sought to explain a causal relationship. The correlational studies, on the other hand, were synthesized separately since the relationship is not causal but associational.

Thus, four types of meta‐analyses were conducted: two based on individuals (experimental and correlational), one based on the relationship between online comments (non‐individual‐based studies), and another based on the relationship between online comments and hate crimes in specific geographic units (ecological studies).

In some experimental studies, more than one experimental condition was used. Apart from the control group and the group exposed to hateful content, in some cases, a third group was included that was exposed to content that could not be qualified as hateful, without being a control group either. In this case, only the control group and the group directly exposed to hateful content were included.

The geographic units need one last precision. These studies have analyzed the influence of comments or posts on social media containing hate speech coming from a specific geographic unit (city, municipality, etc.) on hate crimes committed in that same geographic unit. To make these specific graphical units minimally comparable, the smallest reported geographic unit, e.g., cities, counties, or municipalities, was taken as the reference.

#### 4.3.10 Sensitivity analysis

We applied the “One study removed” technique provided by the Comprehensive Meta‐Analysis (CMA) software to detect and examine possible outliers in each meta‐analysis. This technique is only reliable and informative when the meta‐analyses have at least three studies. Therefore, we only report the results of those meta‐analyses that meet this criterion. We inspected the results to identify whether a single effect size exerted an influence on heterogeneity. To do so, we examined whether the elimination of a single study results in a non‐significant *Q* value.

#### 4.3.11 Deviations from the protocol

Some minor changes were made in relation to the published protocol of this systematic review (Hassan et al., 2022). The protocol indicated for example that the literature search would include studies up to 2020. The current review extended the literature search to studies published until the end of 2021. We also included a new database: Political Science Complete (hosted on EBSCO). A relatively important change refers to the inclusion of studies that are not based solely on individuals. Note here that studies not based on individuals were synthesized independently.

The types of exposure were refined and reclassified into 3 categories already mentioned. For example, the category “consumption” was eliminated as it did not differ substantially from the active search for hate content. It was also clearly stated in the methodology that studies based on direct victimization would be excluded as they are substantially different from simple exposure to this content.

In the published protocol of this systematic review (Hassan et al., 2022), we organized the potential outcomes based on the individual, community, and societal levels. As already mentioned in the section “Types of outcome measures,” we reorganized the outcomes into 5 dimensions or general categories that we consider best fit the reality of the data obtained.

We also decided to change the standard statistic for presenting these outcomes from Fischer's *Z* to Cohen's *d*, since this indicator of effect size is frequently used and easily understood and interpreted.

In the heterogeneity assessment, we included prediction intervals to assess the magnitude of data dispersion. Finally, as an indicator of potential publication bias, we included the Egger regression for its ease of interpretation.

## 5 RESULTS

### 5.1 Description of studies

#### 5.1.1 Results of the search

**Figure 2:**
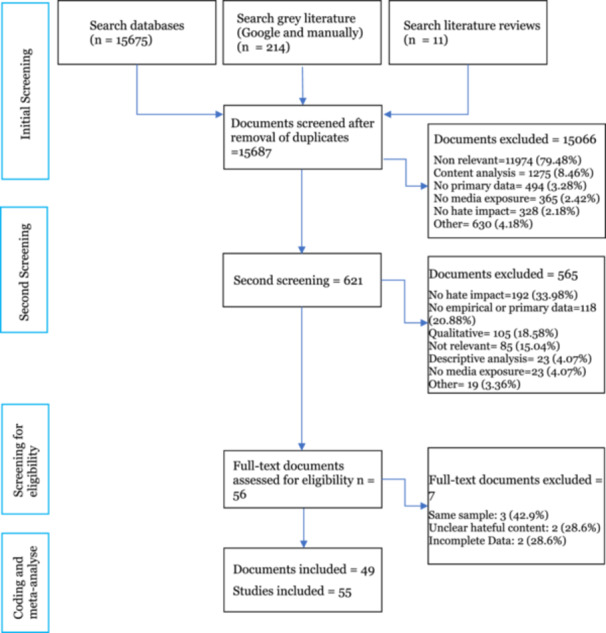
SEQ Figure\* ARABIC 2. Prisma flowchart.

After the selection process, 49 publications comprising 55 studies were retained. The result of this process can be seen in Figure 2. The initial result of the literature search started with 15,675 publications from academic databases, 214 through the gray literature review and Google search, and 11 publications through other similar systematic reviews (Bliuc et al., 2018; Carthy et al., 2020; Castaño‐Pulgarín et al., 2021; Hassan et al., 2018; Waqas et al., 2019; Windisch et al., 2022; Wolfowicz et al., 2022).

After eliminating duplicate documents, a total of 15687 publications were included in the initial review process. As outlined in the methods section, each title and abstract were evaluated by two reviewers, and their agreement was measured using Cohen's *κ* coefficient. Since the resulting coefficient was 0.581, all discrepancies between reviewers were further examined in a second screening phase. If the disagreement persisted, a final decision was made by the principal researchers.

During this second screening phase, a total of 15,066 publications were excluded from the review process. The primary reasons for exclusion were irrelevance to the scope of this systematic review (79.48% of exclusions), reliance on content analysis without empirical data (8.46%), absence of primary or empirical data (3.28%), lack of exposure to hate in a media source (2.42%), or failure to assess the impact of hate exposure (2.18%).

Out of the 621 publications that remained after the initial screening phase, 565 publications were further excluded during the second screening phase. These exclusions were primarily due to the publications not addressing the impact of hate exposure (33.98%), lacking empirical or primary data (20.88%), being qualitative or content analysis studies (18.58%), or not being relevant to the scope of this systematic review (15.04%).

After the two screening phases, a total of 56 publications, which included 67 studies, were considered eligible for inclusion. However, upon reviewing the full text, seven publications were subsequently excluded, along with two of the three studies included in the study conducted by Rieger et al. (2013, Study 1 and Study 2) and the first study by Hameleers (2019). In the dedicated section, we will provide detailed explanations for the exclusion of these specific publications and studies.

In the final stage of this systematic review, data necessary for synthesis were extracted from the 49 publications that fulfilled all the inclusion criteria. To assess the quality of the 55 studies, the Mixed Method Appraisal Tool (MMAT) was applied during this phase.

While all the included studies provided sufficient information for inclusion in a meta‐analysis, only 50 studies contributed to these analyses. This limitation was due to the unavailability of a sufficient number of studies for the calculations required (a minimum of two studies). A list of these non‐contributive five studies can be observed in Table 3.

#### 5.1.2 Included studies

A description of the selected studies can be seen in Table 2. These studies were published between 1996 and 2021, with most of them being published since 2015. As indicated above, this systematic review includes correlational (25 studies) and experimental studies both randomized (22 studies) and non‐randomized (8 studies). Most of these studies provide data extracted from individuals, however, we also decided to include 6 studies that are based on quantitative analysis of comments, posts, or the relationship between both and specific geographic areas.

**Table 2:** Studies retained.

Name citation style APA	*N*	Males%	Age (mean)	Year of data	Exposed group	Hate target	Country	Language	Publication status	Design (ex, quasi‐ex or Correlational)	Type of media
Blaya & Audrin, 2019	1889	49.75	14.63	2016	Nonspecific group	Religion, ethnicity	France	English	Journal article	Correlational	Social media
Cano et al., 2021	200	49	21.3	–	Latinos (minority)	Latinos	USA	English	Journal article	Correlational	Social media
Costello et al., 2017	963	50	24,66	2015	Nonspecific groups	Nonspecific group	USA	English	Journal article	Correlational	Social media
Costello et al., 2019	594	51.8	15‐36	2016	Nonspecific groups	Nonspecific group	USA	English	Journal article	Correlational	Social media
English et al., 2020	101	41	14.5	2014–2015	Black Americans (minority)	Black Americans	USA	English	Journal article	Correlational	Social media
Keipi et al., 2018, Study 1	1014	50.4	23.1	2013	Nonspecific groups	–	USA	English	Journal article	Correlational	Social media
Keipi et al., 2018, Study 2	555	49.9	22.6	2013	Nonspecific groups	–	Finland	English	Journal article	Correlational	Social media
Müller & Schwarz, 2021	4466 municipalities	–	–	2015–2017	Nonspecific group	Nonspecific group	Germany	English	Journal article	Correlational	Social media
Näsi et al., 2015	723	–	15 ‐ 18	2013	Nonspecific groups	–	Finland	English	Journal article	Correlational	Social media
Oksanen et al., 2020, Study 1	2113	46.3	41.61	2015	Nonspecific groups	Nonspecific group	France	English	Journal article	Correlational	Social media
Oksanen et al., 2020, Study 2	1661	48.5	41.51	2015	Nonspecific groups	Nonspecific group	Spain	English	Journal article	Correlational	Social media
Oksanen et al., 2020, Study 3	1003	48.75	47.68	2015	Nonspecific groups	Nonspecific group	Finland	English	Journal article	Correlational	Social media
Oksanen et al., 2020, Study 4	1013	51.5	49.63	2015	Nonspecific groups	Nonspecific group	Norway	English	Journal article	Correlational	Social media
Oksanen et al., 2020, Study 5	1420	45.1	48.1	2015	Nonspecific groups	Nonspecific group	USA	English	Journal article	Correlational	Social media
Pauwels & Schils, 2016	6020	35.3	20.21	2012	Nonspecific groups	Immigrants	Belgium	English	Journal article	Correlational	Social media
Räsänen et al., 2016	723	34.9	16.6	2013	Black Americans (minority)	Nonspecific group	Finland	English	Journal article	Correlational	Social media
Soral et al., 2018, Study 1	1007	48	46.27	–	Nonspecific groups	LGBT, Muslims	Poland	English	Journal article	Correlational	‐‐
Soral et al., 2018, Study 3	682	49.6	16.71	–	Nonspecific groups	Muslims/refugees	Poland	English	Journal article	Correlational	‐‐
TaeHyuk Keum & Hearns, 2022	765	40	20.86	–	American minorities (minority)	Race	USA	English	Journal article	Correlational	Social media
Tynes et al., 2008	264	48	16	–	Nonspecific groups	Ethnicity	USA	English	Journal article	Correlational	Social media
Tynes et al., 2014	627	45.3	14.42	–	Nonspecific groups	Race	USA	English	Journal article	Correlational	Social media
Voigtländer & Voth, 2015	5300	–	–	1996 –2006	Nonspecific groups	Jews	Germany	English	Journal article	Correlational	Political propaganda
Wachs et al., 2019, 2021	6829	49.2	14.93	–	Nonspecific groups	Ethnicity, religion	Cyprus, Germany, Greece, India, South Korea, Spain, Thailand, USA	English	Journal article	Correlational	Social media
Wojcieszak, 2010	114	86	33	2005	Participants in neo‐Nazi online forums	Nonwhite people	USA	English	Journal article	Correlational	Social media
Ybarra et al., 2008	1585	52	13	2006	Nonspecific Groups	Nonspecific group	USA	English	Journal article	Correlational	Social media
Anspach, 2021	883	45.36	35.67	2018	White Americans	Black Americans	USA	English	Journal article	Experimental	Social media
Arendt et al., 2015	186	20.9	25.41	–	Nonspecific groups	Immigrants	Austria	English	Journal article	Experimental	Political propaganda
Botan et al., 2020; Buturoiu & Corbu, 2020	351	–	–	2020	Nonspecific groups	Roma people	Romania	English	Journal article	Experimental	Social media
Brinson, 2010, Study 1	183	–	30	–	Muslims (minority)	Muslims	USA	English	Thesis	Experimental	Traditional media
Brinson, 2010, Study 2	189	–	42	–	Non‐Muslims	‐‐	USA	English	Thesis	Experimental	Traditional media
Chavez et al., 2019	280	10.4	20.8	2016 –2017	Mexican Americans (minority)	Mexicans	USA	English	Journal article	Experimental	Political propaganda
Hameleers, 2019, Study 2	277	47.3	48.52	–	Nonspecific Groups	Refugees\Immigrants	Netherland	English	Journal article	Experimental	Traditional media
Lee‐Won et al., 2020	210	48.1	32.3	–	Black‐Americans (minority)	Black Americans	USA	English	Journal article	Experimental	Social media
Matthes & Schmuck, 2017	199	35	54.6	–	Nonspecific groups	Immigrants\Muslims	Austria	English	Journal article	Experimental	Political propaganda
Newman et al., 2021	997	50.4	33 (median age)	2016	Nonspecific groups	Ethnicity	USA	English	Journal article	Experimental	Traditional media
Obermaier et al., 2021	362	52	33	2020	Muslims (minority)	Muslims	Germany	English	Journal article	Experimental	Social media
Rieger et al., 2013, Study 3	70	100	24.31	2011	Nonspecific groups	Muslims\immigrants	Germany	English	Report	Experimental	Political propaganda
Schmuck & Matthes, 2017	471	47.3	43	–	Nonspecific groups	Muslims\immigrants	Austria	English	Journal article	Experimental	Political propaganda
Schmuck & Matthes, 2019	174	69	45.3	2016	Nonspecific groups	Muslims	Austria	English	Journal article	Experimental	Political propaganda
Schmuck & Tribastone, 2020	143	37	23.53	2018	Muslims (minority)	Muslims	Austria	English	Journal article	Experimental	Political propaganda
Schmuck et al., 2017	145	36	22.78	2016	Muslims (minority)	Muslims	Austria	English	Journal article	Experimental	Political propaganda
Shortland et al., 2022	1112	82.46	18.26	–	Nonspecific groups	Nonspecific group	USA	English	Journal article	Experimental	Social media
Soral et al., 2018, Study 2	75	16	23.21	–	White Polish	Nonspecific group	Poland	English	Journal article	Experimental	Social media
Steele et al., 2015	96	9.4	20.01	–	Mostly Americans Whites	Muslims	USA	English	Journal article	Experimental	Traditional media
Velasco, 2016	95	41	–	–	Nonspecific groups	Latinos	USA	English	thesis	Experimental	Social media
Weber et al., 2020	253	49	43.8	–	Nonspecific groups	Refugees	Germany	English	Journal article	Experimental	Traditional media
Ziegele et al., 2018	497	49	44	–	Nonspecific groups	Refugees\immigrants	Germany	English	Journal article	Experimental	Social media
Dashti et al., 2015	715	36	–	2012	Nonspecific groups	Shiites and Bedouins	Kuwait	English	Journal article	Quasi‐experimental	Traditional media
Gallacher, 2021	33,089,208 messages	–	–	2016–2018	Nonspecific group	Nonspecific group	Nonspecific country	English	Thesis	Quasi‐experimental	Social media
Lee & Leets, 2002	108	57.4	16 (median age)	–	Nonspecific groups	Non‐White people	USA	English	Journal article	Quasi‐experimental	Social media
Nguyen et al., 2021	2344 counties	–	–	2015–2018	Nonspecific groups	Nonspecific group	USA	English	Journal article	Quasi‐experimental	Social media
Relia et al., 2019	100 cities	–	–	2016 –2017	–	Nonspecific group	USA	English	Journal article	Quasi‐experimental	Social media
Saha et al., 2019	5,884,905 comments	–	–	2008–2017	Nonspecific groups	Nonspecific group	USA	English	Journal article	Quasi‐experimental	Social media
Spörlein & Schlueter, 2021	5,152 comments	–	–	2015–2017	Nonspecific groups	Refugees	Germany	English	Journal article	Quasi‐experimental	Social media
Walker‐Matthews, 1996	69	100	27.78	–	Heterosexual and homosexual American men	Sexual orientation	USA	English	thesis	Quasi‐experimental	Traditional media

*Source*: Authors.

Four publications included more than one study or based their measurements on more than one sample, and two studies published in two different papers used the same samples. This brings the total to 55 studies. Two publications by Wachs et al., (2019, 2021) that use the same sample and have the same objectives were included as they each present different associations that are relevant to our review. The same situation occurs in the case of Botan et al. (2020) and Buturoiu and Corbu (2020). The studies conducted by Keipi et al. (2018) and Oksanen et al. (2020) are international comparisons with samples taken independently by different researchers in different countries and in which, the information could be fully extracted by country. This made it possible to include these samples independently in this systematic review. A similar situation occurred in the case of the studies by Wachs et al. (2019, 2021), however, these samples could not be separated so they were included as a single large sample within our review.

This systematic review includes studies with a wide range of participants, settings, and types of exposure to hate which may account for the significant heterogeneity observed in the meta‐analysis results. In terms of sample sizes, the studies include samples ranging from 69 to 6829 participants. Correlational studies encompass sample sizes ranging from 101 to 6829 participants, while experimental and quasi‐experimental studies involve participant numbers between 69 and 1112. On the other hand, studies that were not based on individuals span a wide range, from studies centered on 5152 comments on YouTube videos (Spörlein & Schlueter, 2021) to extensive analyses of 33,089,208 messages on the GAB online platform (Gallacher, 2021). Ecological studies examining the relationship between comments and specific regions incorporate data from 100 cities in the United States (Relia et al., 2019) to 4466 municipalities in Germany (Müller & Schwarz, 2021). Most studies primarily draw samples from the United States (26), Germany (8), and Austria (6), with only two studies incorporating participants from non‐Western countries.

Among the analyzed studies, six studies focusing on individuals did not provide information about the gender distribution of participants, while four studies did not report the age of the participants. However, in the remaining correlational studies, there was a balanced ratio of male and female participants (average male = 48.65%), except for one study (Wojcieszak, 2010) where the percentage of male participants was notably higher. The average age of participants in these studies was relatively young (26.2), although some studies included samples with an average age above 40 years (Oksanen et al., 2020; Soral et al., 2018, Study 1). For both randomized and non‐randomized experimental studies, the average percentage of male participants was 47.2%, although there were studies where only men were included (Rieger et al., 2013, Study 3; Walker‐Matthews, 1996) and others where the percentage of female participants was significantly higher (Chavez et al., 2019; Soral et al., 2018, Study 2). The mean age in experimental studies was relatively higher compared to correlational studies (32.3), and there were studies where the median age was 16 (Lee & Leets, 2002) or the average age was 48.52 (Hameleers, 2019, Study 2).

In most cases, the exposure to hate content occurred within an online or social media context (37 studies), while only 8 studies reported such exposure in traditional media platforms. It is worth noting that even in these traditional media studies, a significant portion of the content originated from or was influenced by online sources. Alternatively, in the remaining studies, the exposure to hate content was delivered through political propaganda, primarily associated with extreme right‐wing groups.

As we indicated in the methodological section, we have determined three types of interaction with this content: “exposure to,” “active search for,” and “participation” in hate speech forums. In 53 of the 55 retained studies, participants were exclusively exposed to hate content, while in only one study, participants actively searched for hate content, and in another study, participants actively participated in forums where this content was shared.

Regarding the composition of the exposed groups, most instances involved either a nonspecific group or individuals belonging to the majority social group. Specifically, three experimental studies focused exclusively on exposing hate content to a group of white individuals. However, in 10 studies, the exposed group was clearly identified as a minority, with four studies employing a correlational design and the remaining studies utilizing experimental or quasi‐experimental designs. Concerning the groups targeted by the hate speech to which participants were exposed, 16 of the studies used content that did not target any specific group, 15 used content directed at a religious group, with the vast majority targeting Muslims (12); 14 studies exposed content related to ethnic groups; 12 studies used content directed at immigrants and two at LGBTQ groups. Four studies did not reference the group targeted by the exposure content, and six studies presented more than one specific group.

#### 5.1.3 Excluded studies

As mentioned in the section on search results, seven eligible publications and three studies included in two publications originally retained were finally excluded from this analysis. In half of them, the samples included were the same as in other studies and/or did not present associations relevant to this systematic review. The Wachs and Wright (2018) study is, for example, based on the same German sample that was used in the eight‐country comparative study by Wachs et al. (2019) in which the relationship between witnessing hate speech online and the perpetration of hate speech was analyzed. The Kaakinen et al. (2018) study used the same sample as the Oksanen et al. (2020) study and analyzed the same association between exposure to online hate and perceived fear. We decided to retain the latter publication because it was more complete. The study of Frischlich et al. (2015) evaluates the influence of mortality salience on the persuasiveness and interest aroused by extremist propaganda videos. These videos are the same ones used in the Rieger et al. (2013) study, which we have included in our systematic review. The Frischlich et al. (2015) study does not present data concerning a direct association between hate exposure and its impacts and the sample appears to be from the same sample as the Rieger et al. (2013) study.

The other publications were excluded because the relationship between exposure to hate and its consequences was unclear, because the study used an inappropriate design for this systematic review, or because the data were incomplete. This is the case of the study by Bail et al. (2018) which analyzes the relationship between active search for anti‐Muslim content online and radicalization. Although this study may be of interest for this systematic review, the anti‐Muslim content sought may not be exclusively hate speech according to our criteria and may also include simple negative perceptions that are not necessarily hateful. The study by Bäck et al. (2018) analyzed online comments in a xenophobic and anti‐immigration forum to analyze identity formation processes. This forum, as the authors mention, involves both hate speech and anti‐hate speech groups and Bäck et al. (2018) did not exclusively select hate speech in their analyses, so it is not possible to establish a clear and direct relationship between exposure to hate speech and its consequences. In 2001, Leets conducted a quasi‐experimental study that analyzed individuals' reactions to hate websites and investigated the degree to which these websites are considered offensive. Unfortunately, Leets did not use any comparison group or compare the same group at different times, so were unable to include the results in this research. The study by He et al. (2021) analyzes the spread of hate speech against Asians on Twitter, that is, how a hateful comment could influence the appearance of a new hateful comment. However, the publication presents only one graph that accounts for this relationship and the authors did not respond to a request for additional information.

Finally, three studies sited in two publications included in this systematic review were excluded (Hameleers, 2019, Study 1; Rieger et al., 2013, Study 1 and Study 2). The report by Rieger et al. (2013), includes three focus research studies of which we only included the third study since only this latter met our inclusion criteria. The two initial studies were excluded as the first one analyzes only the content of hate speech and the second one, despite experimentally exposing individuals to this type of content, does not make any comparisons relevant to this systematic review, that is, a comparison before and after exposure or between a control group and an exposed group. Of the two studies presented by Hameleers (2019, Study 1), we excluded the first one, as it was only a content analysis.

### 5.2 Risk of bias in included studies

In the following section, we will provide a concise overview of the findings from the evaluation of the methods employed in the selected studies. For a comprehensive and detailed assessment, please refer to Supporting Information S1: Appendix C of this study. It is important to note that this evaluation pertains to all the studies retained in this systematic review, not solely those included in the meta‐analysis.

#### 5.2.1 Randomized controlled trials

Out of the total number of experimental studies analyzed, only eight were deemed to have correctly executed the randomization process. Although all the studies mentioned that participants were randomly assigned to either the control group or the group exposed to hate content, very few provided clear details on how this randomization was conducted, which is a crucial criterion in the methodological evaluation tool utilized. Likewise, in most studies (13), it was not possible to ascertain whether the control and treatment groups were comparable at the baseline due to a lack of specific data. Only nine studies reported this information, which is typically provided to confirm the proper execution of randomization.

In contrast, in all the experimental studies included in this systematic review (22), a significant majority of participants contributed to nearly all measures. To meet this methodological evaluation criterion, we established a benchmark that required at least 80% of participants to contribute to all measures, and this threshold was widely surpassed in almost all studies. This can be attributed, in part, to the participants' high level of adherence to the experimental conditions (22), which is the final criterion employed in the evaluation of experimental studies in this systematic review.

#### 5.2.2 Quantitative non‐randomized studies

The category “Quantitative non‐randomized studies” in the MMAT encompasses a wide range of studies that do not fall under the experimental or quantitative‐descriptive categories. As a result, all retained correlational and quasi‐experimental studies were evaluated within this category.

Among the criteria used to assess nonrandomized studies, representativeness emerged as the weakest indicator. In half of the studies (10), it was not possible to determine whether the sample was representative of the target population based on the provided information. In some cases (6), it was evident that the sample was clearly non‐representative.

On the other hand, the evaluation of other criteria yielded positive results. All the studies (33) employed appropriate measures to assess both the outcomes and the exposure. This entailed clear definitions of variables that were well justified, demonstrating acceptable validity and reliability. In nearly all studies (32), the results were complete, indicating that most participants contributed to almost all the outcomes. The consideration of confounding factors was also evident in the retained studies (28). Furthermore, in all studies (33), the exposure to hate content was clearly identified, enabling a comprehensive assessment of its potential impacts.

### 5.3 Synthesis of results

In total, we found 101 effect sizes of association or causality relationships between exposure to hate content and its potential outcomes. These relationships can be classified into 43 different outcomes. Table 3 presents the distinct categories of outcomes that were identified along with their respective definitions that were utilized as the foundation for the meta‐analytical synthesis. These categories are organized based on the type of design in which they were identified. Furthermore, as mentioned in the method section, these categories have been classified into five overarching dimensions: Attitudinal changes (11 outcomes), Intergroup dynamics (11 outcomes), Interpersonal behavior (9 outcomes), Political beliefs (2 outcomes), and Psychological effects (10 outcomes).

**Table 3:** Effect size categories.

Dimension	Category	Outcome	Definition	Experimental studies	Correlational studies	Non‐individual‐based or ecological studies
Attitudinal changes	Attitudes to content	Attitude that supports hate content	Attitude toward distilled hate content	Lee & Leets, 2002; Newman et al., 2021		
	Attitudes toward Outgroups	Explicit negative attitudes	Explicit negative attitudes toward groups with protected characteristics	Anspach, 2021; Brinson, 2010, Study 2; Matthes & Schmuck, 2017; Schmuck & Matthes, 2017; Schmuck & Matthes, 2019; Steele et al., 2015; Weber et al., 2020b; Arendt et al., 2015	Soral et al., 2018, Study 3; Voigtländer & Voth, 2015c	
		Negative stereotypes	Negative stereotypes of groups with protected characteristics	Anspach, 2021; Buturoiu & Corbu, 2020 (Botan et al., 2020); Arendt et al., 2015; Hameleers, 2019, Study 2; Lee‐Won et al., 2020; Matthes & Schmuck, 2017; Schmuck & Matthes, 2017; Schmuck & Matthes, 2019; Weber et al., 2020		
		Perceived external attitudes	Perceptions of outgroup attitudes toward the ingroup	Brinson, 2010, Study 1;		
		Positive attitudes	Positive attitudes toward groups with protected characteristics	Botan et al., 2020; Velasco, 2016; Weber et al., 2020 (Buturoiu & Corbu, 2020)		
		Positive attitudes toward supporting minority groups	Positive attitudes for prosocial behaviors toward people with the protected characteristics	Ziegele et al., 2018		
	Attitudes toward freedom of expression	Support for freedom of expression	Support for general freedom of speech	Dashti et al., 2015		
	Personal attitudinal changes	Implicit assessment of terrorism	Evaluation of implicit word associations with the concept of terrorism	Rieger et al., 2013, Study 3		
		Religious identification	Degree of religious identification of Muslims exposed to hate	Schmuck et al., 2017		
	Support for violence	Support for nonviolent action	Support for nonviolent collective action	Schmuck & Tribastone, 2020		
		Support for political violence	Support for violence for political purposes or against groups with protected characteristics	Schmuck & Tribastone, 2020; Shortland et al., 2022		
Intergroup dynamics	Collective identity	Collective identity	National collective identity	Dashti et al., 2015		
		Collective self‐esteem	Perceived collective esteem	Brinson, 2010, Study 1		
	Discrimination	Perceived discrimination	Perception of being discriminated	Lee‐Won et al., 2020; Schmuck & Tribastone, 2020; Schmuck et al., 2017		
	Group boundaries	Permeability of intergroup boundaries	Perceived permeability of intergroup social boundaries	Brinson, 2010, Study 1;		
	Social norms	Knowledge of social norms	Level of knowledge of social norms		Soral et al., 2018, Study 3	
		Perceived difficulty of supporting minorities	Perceived level of difficulty with prosocial behaviors toward individuals with protected characteristics	Ziegele et al., 2018		
		Perception of social pressure on supporting minorities	Perceived social pressure on prosocial behavior toward individuals with protected characteristics	Ziegele et al., 2018		
	Social trust	General social trust	General level of trust in others		Näsi et al., 2015	
		Intergroup trust	Level of trust between groups (Muslim vs. non‐Muslim)	Brinson, 2010, Study 1; Brinson, 2010, Study 2		
		Interpersonal trust	Interpersonal trust with non‐intimate individuals (acquaintances, colleagues)		Näsi et al., 2015	
		Online interpersonal trust	Trust in online acquaintances		Näsi et al., 2015	
Interpersonal behavior	Offline behavoir	Outgroup discrimination intent	Statement of intent to discriminate against a group because of its protected characteristics	Soral et al., 2018, Study 2	Soral et al., 2018, Study 1; Soral et al., 2018, Study 3	
		Externalizing Behavior	Observable actions or behaviors that involve disruptive, aggressive, or rule‐breaking behaviors		Tynes et al., 2014	
		Hate crime	Offline hate crime rate			Müller & Schwarz, 2021; Nguyen et al., 2021; Relia et al., 2019
		Prosocial behavior toward minorities	Prosocial behaviors toward people with protected characteristics		Ziegele et al., 2018	
		Violent behavior	Violent behavior against property/people		Pauwels & Schils, 2016; Ybarra et al., 2008	
	Online behavoir	Hate contagion	The propagation or transmission of hate speech from one individual or group to another			Gallacher, 2021; Spörlein & Schlueter, 2021
		Online perpetration	The publication, sharing or forwarding of humiliating or hateful messages, images or comments about a specific group of people on the Internet	Obermaier et al., 2021	Blaya & Audrin, 2019; Wachs et al., 2019 (2021)	
		Online victimization	Being the target of online hate attacks because of protected characteristics		Costello et al., 2017; Wachs et al., 2019 (2021); Räsänen et al., 2016	
		Resistance to hate speech	Degree of resistance/opposition to hateful content	Lee & Leets, 2002; Obermaier et al., 2021		
Political beliefs	Political views	Extreme views	Degree of agreement with extreme ideological statements		Wojcieszak, 2010	
		Political support for the right‐wing party	Voting intention for the right‐wing party	Schmuck & Matthes, 2019		
Psychological effects	Emotional reactions to content	Aversion	Negative emotional reaction associated with the content of the message	Soral et al., 2018, Study 2; Obermaier et al., 2021	Costello et al., 2019; Soral et al., 2018, Study 1; Soral et al., 2018, Study 3	
		Content Anxiety	Symptomatic anxiety reaction associated with hate content.		Cano et al., 2021; Tynes et al., 2008; Tynes et al., 2014;	
		Online stress expression	Online stress symptoms in social media users exposed to hate content			Saha et al., 2019
	Emotional reactions to Exo‐groups	Negative emotional reaction toward groups with protected characteristics.	Negative emotional reaction to groups with protected characteristics	Chavez et al., 2019; Matthes & Schmuck, 2017; Schmuck et al., 2017; Velasco, 2016		
		Positive emotions toward groups with protected characteristics	Positive emotional reaction to groups with protected characteristics	Chavez et al., 2019		
		Relational anxiety	Symptomatic anxiety reaction when interacting with individuals with protected characteristics	Matthes & Schmuck, 2017; Schmuck & Matthes, 2017		
	Personal emotional reactions	Depression	Depression symptoms	Lee‐Won et al., 2020; Walker‐Matthews, 1996	Tynes Cano et al., 2021; English et al., 2020; et al., 2008; TaeHyuk Keum & Hearns, 2022; Tynes et al., 2014	
		Satisfaction with life	Level of personal life satisfaction		Keipi et al., 2018, Study 1; Tynes et al., 2008a; Keipi et al., 2018, Study 2	
		Self‐esteem	Perceived self esteem	Schmuck et al., 2017		
		Social Fear	Perception of social fear after a terrorist attack		Oksanen et al., 2020, Study 1; Oksanen et al., 2020, Study 2; Oksanen et al., 2020, Study 3; Oksanen et al., 2020, Study 4; Oksanen et al., 2020, Study 5	

*Source*: Authors.

This taxonomy of outcomes was developed through an inductive process in which we first identified all the outcomes reported by the studies, as well as their definitions. Based on this information, the outcomes that measure the same underlying construct, as indicated in the methodological section, were coded with the same name. With this initial list of coded outcomes, a second level of synthesis was conducted to group them into broader categories. Finally, these broader categories were further regrouped according to the five dimensions mentioned in the previous paragraph.

We have conducted a comprehensive synthesis of the relationship between exposure to hate and its impact on various outcomes through 24 distinct meta‐analyses (Table 4). These analyses comprise 12 meta‐analyses of experimental and quasi‐experimental studies, 10 meta‐analyses of correlational studies, one meta‐analysis of non‐individual‐based studies and one meta‐analysis of ecological studies. Nineteen outcomes could not be synthesized by meta‐analysis as only one study was identified for each of them, including the two outcomes of the “Political beliefs” dimension. These outcomes are presented in Table 3 and can be readily identified by the presence of only one reference.

**Table 4:** Number of studies included in each meta‐analysis.

Dimension	Outcome	Number of studies included per meta‐analysis		
		Experimental studies	Correlational studies	Non‐individual‐based or ecological studies
Attitudinal changes	Attitude that supports hate content	2		
	Explicit negative attitudes	8	2	
	Negative stereotypes	9		
	Positive attitudes	3		
	Support for political violence	2		
	Perceived discrimination	3		
	Intergroup trust	2		
Interpersonal behavior	Outgroup discrimination intent		2	
	Hate crime			2
	Violent behavior		2	
	Hate contagion			2
	Online perpetration		2	
	Online victimization		3	
	Resistance to hate speech	2		
Psychological effects	Aversion	2	3	
	Content Anxiety		3	
	Negative emotional reaction toward groups with protected characteristics.	4		
	Relational anxiety	2		
	Depression	2	5	
	Satisfaction with life		3	
	Social Fear		5	

As previously explained, this systematic review primarily focuses on two types of relationships: causal relationships established through meta‐analysis of experimental and quasi‐experimental studies, and associations between variables examined through meta‐analysis of correlational studies. In the latter case, we aimed to synthesize non‐causal associations between exposure to hate and other variables.

We decided to conduct separate syntheses for studies primarily centered on social media comments, as these studies do not focus on individual analysis or experiences. The subsequent meta‐analyses will address two specific areas. The first examines the contagion of hate between comments or posts, while the second explores the association between hate speech on social networks and hate crimes within specific regions.

The meta‐analysis prioritized using the full effects between hate exposure and outcomes. However, when this was not possible, partial effects were used. A summary of this information can be found in Supporting Information S1: Appendix D. In the case of the meta‐analyses of the experimental studies, we were able to use the full effects in 8 of the 10 estimates. In contrast, we were able to use the full effects in only two of the meta‐analyses of the correlational studies and in none of the meta‐analyses of the non‐individual‐based or ecological studies. In the cases in which it was possible to use the complete effect, it was due to the supplementary information requested from the authors of these studies.

In what follows, we will present the results of this systematic review by organizing the information according to the five main dimensions. When possible, the results of experimental and correlational studies that are related to the same outcome will be presented at the same time.

#### 5.3.1 Attitudinal changes

Attitudinal changes refer to changes in individuals' attitudes, beliefs, or opinions toward a particular content or group in relation to or caused by exposure to hate. We were able to identify 10 outcomes through this systematic review and conduct 6 meta‐analyses, 5 of which address causal questions about the impact of hate exposure on attitudinal changes. These meta‐analyses address attitudes toward hate content and toward the outgroup as well as support for political violence. Five other outcomes related to attitudinal changes were identified but could not be synthesized through meta‐analysis due to a lack of sufficient studies (Table 5).

**Table 5:** Meta‐analysis on attitudinal changes related to exposure to hate content.

Outcome	Design	Std diff in means	95% CI	*Q*	*I* ^2^	*τ* ^2^	PI	*N* (*k*)
Attitudes that support hate content	Ex	1.932	−0.831, 4.695	75.479***	98.675	3.921	NA	386 (2)
Explicit negative attitudes	Ex	0.414*	0.005, 0.824	109.583***	93.61	0.316	−1.054, 1.882	1803 (8)
	Corr	0.322***	0.14, 0.504	4.962*	79.846	0.014	NA	4968 (2)
Negative stereotypes	Ex	0.28+	−0.018, 0.586	75.783***	89.44	0.185	−0.796, 1.364	1932 (9)
Positive attitudes	Ex	−0.227+	−0.466, 0.011	2.107	5.087	0.003	−1.904, 1.45	294 (3)
Support for political violence	Ex	0.081	−0.122, 0.284	0.002	0	0	NA	373 (2)
*Outcomes not included in meta‐analysis*								
Implicit assessment of terrorism	Ex	1.1744***	0.557, 1.792	–	–	–	–	28 (1)
Perceived external attitudes	Ex	−0.177	−1.170, 0.390	–	–	–	–	122 (1)
Religious identification	Ex	−0.6274	−1.155, 1.035	–	–	–	–	145 (1)
Support for freedom of expression	Ex	−5.525***	5.848, −5.202	–	–	–	–	715 (1)
Support for nonviolent action	Ex	−0.0902	−0.497, 0.317	–	–	–	–	93 (1)

Abbreviations: 95% CI, 95% lower and upper confidence intervals; Corr, correlational study; Ex, experimental design; *I* ^2^, statistics for the proportion of variation across studies attributed to heterogeneity; *k*, number of studies; *N*, total sample size; PI, prediction interval; *Q*, Cochran's *Q* statistic for heterogeneity and *χ* ^2^ test; *τ* ^2^, variance of true effect size.

***<0.001; *<0.05; ^+^<0.10.

*Source*: Authors.

These meta‐analyses suggest that exposure to hate content leads to changes in attitudes toward groups that have been targeted by such content. Specifically, exposure to hate content leads to an increase in explicit negative attitudes toward these groups, with moderate estimates found in both experimental and correlational studies (*d*_Ex_ = 0.414; 95% CI = 0.005, 0.824; *p* < 0.05 and *d*_corr_ = 0.322; 95% CI = 0.14, 0.504; *p* < 0.01). Two other outcomes appear marginally significant. Exposure to hate potentially decreases positive attitudes toward groups that have been targeted by this content (*d*_exp_ = −0.227; 95% CI = − 0.466, 0.011; *p* < 0.1) and potentially increases negative stereotypes regarding these specific groups (*d*_exp_ = 0.28; 95% CI = − 0.018, 0.586; *p* < 0.1). In both cases, the effect size is small. In contrast, exposure to hate content does not significantly influence changing attitudes toward the content itself and does not significantly increase support for political violence. However, these latter results should be interpreted with caution because we were able to identify only two studies per outcome that addressed these issues.

These results should also be interpreted with caution, as they present some weaknesses in the assessment of the risk of biases. In the experimental studies, the equivalence of comparison groups at baseline was not confirmed, and the correlational studies included in the meta‐analysis did not control for confounding variables. As a result, we cannot be completely confident in these findings.

##### 5.3.1.1 Outcomes not included in meta‐analysis

In addition to the previously discussed results, five other outcomes were examined, each represented by a single study, which also requires cautious interpretation. Specifically, exposure to hate content significantly decreased general support for freedom of expression (*d*_exp_ = –5.525; 95% CI = −5.848, −5.202; *p* < 0.001). Likewise, a significant increase was observed in the negative implicit evaluation of terrorism; that is, participants showed stronger implicit associations between terrorism and negatively valued content after being exposed to hate content (*d*_exp_ = 1.174; 95% CI = 0.557, 1.792; *p* < 0.001). Other results showed non‐significant effects. Exposure to hate content was associated with a non‐significant decrease in perceptions of outgroup attitudes toward the ingroup, a non‐significant decrease in the degree of religious identification among Muslims exposed to hate, and a non‐significant decrease in support for nonviolent collective action.

#### 5.3.2 Intergroup dynamics

Intergroup dynamics refer to the various processes and relationships that affect how social groups interact and perceive each other, and the factors that influence the level of trust and the attitudes toward different groups within a community. This is particularly relevant in the context of relations between groups that are the target of hate speech and majority groups. Consequently, the repercussions of exposure to hate content on these variables may have a bearing on intergroup relations at the societal and communal levels. We identified 9 outcomes, of which only 2 meta‐analyses could be carried out in association with experimental studies (Table 6).

**Table 6:** Meta‐analysis on intergroup dynamics related to exposure to hate content.

Outcome	Design	Std diff in means	95% CI	*Q*	*I* ^2^	*τ* ^2^	PI	*N* (*k*)
Intergroup trust	Ex	−0.308**	−0.559, −0.058	0.107	0	0	NA	248 (2)
Perceived discrimination	Ex	0.346	−2.98, 0.989	17.997***	88.887	0.287	−7.636, 8.32	355 (3)
*Effect sizes not included in meta‐analysis*								
Collective identity	Ex	1.1444***	0.985, 1.304	–	–	–	–	715 (1)
Collective self‐esteem	Ex	−0.0139	−0.369, 0.341	–	‐‐		–	122 (1)
General social trust	Corr	−2.903***	−3.119, −2.687	–	–	‐‐	–	723 (1)
Interpersonal trust	Corr	−1.425***	−1.597, −1.253	–		–	‐‐	723 (1)
Knowledge of social norms	Corr	0.4945***	0.342, 0.647	–	–	–	‐‐	682 (1)
Online interpersonal trust	Corr	1.485***	1.312, 1.658	–	–	–	–	723 (1)
Permeability of intergroup boundaries	Ex	−0.3182	−0.675, 0.039	–	–	–	–	122 (1)

Abbreviations: 95% CI, 95% lower and upper confidence intervals; Corr, correlational study; Ex, experimental design; *I* ^2^, statistics for the proportion of variation across studies attributed to heterogeneity; *k*, number of studies; *N*, total sample size; PI, prediction interval; *Q*, Cochran's *Q* statistic for heterogeneity and *χ* ^2^ test; *τ* ^2^, variance of true effect size.

***<0.001; **<0.01.

*Source*: Authors.

According to the studies included in these meta‐analyses, exposure to hate content causes a moderate decrease in trust between groups that are the target of hate speech and majority groups (*d*_exp_ = −0.308; 95% CI = −0.559, −0.058; *p* < 0.05). These results are based on two experimental studies that were published by the same author (Brinson, 2010, Study 1 and 2), using independent samples. It would be advisable to have a larger number of studies addressing this relationship to confirm these findings. However, both studies were evaluated positively using our risk of bias assessment tool, suggesting that their results are quite reliable. Conversely, exposure to hate content does not significantly influence the perception among minority groups with protected characteristics regarding being discriminated against because of these characteristics.

##### 5.3.2.1 Outcomes not included in meta‐analysis

Five individual studies that were not synthesized showed statistically significant results. Specifically, exposure to hate content in an experimental study, significantly increased collective identity, that is, individuals' identification with their social group (*d*_exp_ = 1.144; 95% CI = 0.985, 1.304; *p* < 0.001). On the other hand, exposure to hate content was significantly associated with greater knowledge of social norms (*d*_corr_ = 0.495; 95% CI = 0.342, 0.647; *p* < 0.001). However, it was also observed that this exposure was associated with significant decreases in general social trust (*d*_corr_ = −2.903; 95% CI = −3.119, −2.687; *p* < 0.001) and interpersonal trust (*d*_corr_ = −1.425; 95% CI = −1.597, −1.253; *p* < 0.001), indicating that exposure to hate content may be related to an erosion of trust among individuals and within society in general. Paradoxically, in the same study (Näsi et al., 2015) was found that exposure to hate content was associated with a significant increase in online interpersonal trust (*d*_corr_ = 1.485; 95% CI = 1.312, 1.658; *p* < 0.001), possibly due to the formation of like‐minded communities.

Other results showed non‐significant effects. Exposure to hate content was associated with a non‐significant decrease in collective self‐esteem—the value that individuals attribute to their group—and in the permeability of intergroup boundaries, which refers to the ease with which individuals can move between different social groups.

#### 5.3.3 Interpersonal behavior

The behavioral changes that may result from exposure to hate are one of the key questions addressed in our review. In this systematic review, we identified nine outcomes related to this relationship, of which seven meta‐analyses could be performed. However, all these meta‐analyses were conducted with only two studies each, except for online victimization, so their results should be interpreted with caution. Four of these meta‐analyses explored the association between exposure to hate and an outcome through correlational studies, and only one meta‐analysis of experimental studies tested the impact of exposure to hate on the development of hate‐resistant behavior. Finally, two studies based on social media analysis examined the ability of hate comments to propagate into other online comments and the influence that exposure to hate may have on the incidence of hate crimes. Only three outcomes could not be synthesized (Table 7).

**Table 7:** Meta‐analysis on Interpersonal behavior related to exposure to hate content.

Outcome	Design	Std diff in means	95% CI	*Q*	*I* ^2^	*τ* ^2^	PI	*N* (*k*)
Outgroup discrimination intent	Corr	0.145	−0.536, 0.827	47.07***	97.876	0.237	NA	1689 (2)
Online perpetration	Corr	0.363+	−0.028, 0.754	57.56***	98.263	0.078	NA	8718 (2)
Online victimization	Corr	0.721***	0.472, 0.97	30.723***	93.49	0.045	−2.413, 3.854	8515 (3)
Violent behavior	Corr	0.47***	0.328, 0.612	6.213*	83.906	0.009	NA	7602 (2)
Resistance to hate speech	Ex	0.063	−0.754, 0.881	7.424***	86.53	0.303	NA	272 (2)
Hate contagion	NBI	0.509*	0.034, 0.984	3.052+	67.233	0.088	NA	11,035,755 (2)
Hate crimes	Ecol	−0.016	−0.094, 0.063	2.069	51.65	0.002	NA	480,964 (2)
*Outcomes not included in meta‐analysis*								
Externalizing Behavior	Corr	0.7***	0.53, 0.86	–	–	–	–	627 (1)
Online perpetration	Ex	0.148	−0.241, 0.537	–	–	–	–	102 (1)
Outgroup discrimination intent	Ex	−0.469*	−0.928, −0.010	–	–	–	–	75 (1)

Abbreviations: 95% CI, 95% lower and upper confidence intervals; Corr, correlational study; Ex, experimental design; *I* ^2^, statistics for the proportion of variation across studies attributed to heterogeneity; *k*, number of studies; *N*, total sample size; NBI = non‐individual‐based study; PI, prediction interval; *Q*, Cochran's *Q* statistic for heterogeneity and *χ* ^2^ test; *τ* ^2^, variance of true effect size.

***<0.001; *<0.05; ^+^<0.10.

*Source*: Authors.

Meta‐analyses conducted on correlational studies reveal a moderate positive association between exposure to hate content and the manifestation of violent behavior in offline settings (*d*_corr_ = 0.47; 95% CI = 0.328, 0.612; *p* < 0.01). It is important to note that due to the correlational nature of these studies, causality cannot be inferred regarding the relationship between hate exposure and violent behavior. Furthermore, a moderate but marginally significant correlation was observed between exposure to hate content and engagement in online perpetration of hate speech (*d*_corr_ = 0.36; 95% CI = ‐0.028, 0.754; *p* < 0.1). However, the available studies do not provide sufficient evidence to establish a connection between this exposure and the explicit intention to discriminate against individuals with protected characteristics. Conversely, being exposed to hateful content is strongly and positively related to the experience of being a victim of hate speech online (*d*_corr_ = 0.721; 95% CI = 0.472, 0.97; *p* < 0.01). The sole meta‐analysis based on experimental studies did not yield conclusive findings regarding a causal relationship between exposure to hate content and the development of resistance against such content.

Despite the limited number of studies per meta‐analysis, their results are generally reliable according to the risk of bias assessment, except for Online perpetration, for which the studies did not determine whether the samples were representative of the target population.

The studies included in the last two meta‐analyses did not use individuals as the unit of analysis. Instead, they focused on the correlation between online comments or posts, and the occurrence or absence of hate crimes in specific geographical regions (ecological study). Hate contagion, referring to the propensity of an online comment or post to propagate to other comments or posts, exhibits a moderately positive estimate (*d*_nbi_ = 0.51; 95% CI = 0.034–0.984; *p* < 0.05), indicating a significant likelihood of subsequent similar comments following such online expressions. In this case, the two included studies present significant risks of bias, particularly regarding the absence of analysis on the influence of confounding variables and the inability to determine whether the samples were representative of the target population. Notably, the presence of online hate comments does not appear to be significantly linked to the occurrence of hate crimes within the corresponding geographic units^2^ where these expressions were made.

##### 5.3.3.1 Outcomes not included in meta‐analysis

Additionally, three outcomes based on a single study each were identified, which could not be included in a meta‐analysis. First, a strong and significant positive association was found between exposure to hate content and externalizing behavior, which is related to aggressive conduct or the violation of social norms (*d*_corr_ = 0.7; 95% CI = 0.53, 0.86; *p* < 0.001). The other two outcomes were evaluated through experimental studies that examined associations previously described in the meta‐analyses of correlational studies, but which partially contradict those findings. Specifically, the impact of exposure to this type of content on the intention to discriminate against the outgroup showed a moderate and significant effect in a negative direction (*d*_exp_ = –0.469; 95% CI = −0.928, −0.010; *p* < 0.05), indicating a decrease in discriminatory intentions. On the other hand, exposure to hate content did not show a significant effect on the online perpetration of hate speech.

#### 5.3.4 Political beliefs

This category encompasses changes in the political beliefs or ideas of individuals or groups that are related to or caused by exposure to hate content.

##### 5.3.4.1 Outcomes not included in meta‐analysis

Only one experimental study was found that could not be meta‐analyzed (Schmuck & Matthes, 2019). This study investigated how exposure to hate content affects political support for a right‐wing party. The results showed a small and non‐significant effect size (Table 8).

**Table 8:** Political beliefs related to exposure to hate content.

Outcome	Design	Std diff in means	95% CI	*Q*	*I* ^2^	*τ* ^2^	PI	*N* (*k*)
*Outcomes not included in meta‐analysis*								
Political support for the right‐wing party	Ex	0.120	−1.096, 1.336	–	–	–	–	174 (1)

Abbreviations: 95% CI, 95% lower and upper confidence intervals; Corr, correlational study; Ex, experimental design; *I* ^2^, statistics for the proportion of variation across studies attributed to heterogeneity; *k*, number of studies; *N*, total sample size; PI, prediction interval; *Q*, Cochran's *Q* statistic for heterogeneity and *χ* ^2^ test; *τ* ^2^, variance of true effect size.

*Source*: Authors.

#### 5.3.5 Psychological effects

The psychological effects of exposure to hate on individuals are one of the most addressed topics in the included studies. We were able to identify 10 different outcomes, which allowed us to perform nine different meta‐analyses. Only three outcomes could not be synthesized due to a lack of studies. Five of the meta‐analyses tested associations, and four examined the causality between exposure to hate and the emotional reactions described below (Table 9).

**Table 9:** Meta‐analysis on psychological effects related to exposure to hate content.

Outcome	Design	Std diff in means	95% CI	*Q*	*I* ^2^	*τ* ^2^	PI	*N* (*k*)
Aversion	Ex	0.031	−1.219, 1.280	19.918***	94.979	0.773	NA	239 (2)
	Corr	−0.323	−1.015, 0.369	146.245***	98.632	0.369	−9.253, 8.607	2589 (3)
Content anxiety	Corr	0.195	−0.059, 0.45	7351*	72.792	0.036	−2.735, 3.126	1067 (3)
Depression	Ex	1.105***	0.797, 1.423	0.351	0	0	NA	187 (2)
	Corr	0.271*	0.011, 0.53	27.065***	85.221	0.071	‐0.676, 1.217	1933 (5)
Negative emotional reaction toward groups with protected characteristics	Ex	0.014	−2.283, 0.31	8.926*	66.39	0.058	−1.213, 1.24	580 (4)
Relational anxiety	Ex	0.92	−0.289, 2.129	32.064***	96.881	0.737	NA	388 (2)
Satisfaction with life	Corr	−0.186***	−0.279, −0.093	0.147	0	0	NA	1809 (3)
Social fear	Corr	0.206***	0.147, 0.264	6.197	35.451	0.002	0.047, 0.364	7200 (5)
*Outcomes not included in meta‐analysis*								
Online stress expression	NBI	0.4***	0.391, 0.409	–	–	–	–	217,109 (1)
Positive emotional reaction to groups with protected characteristics	Ex	0.2451	−0.043, 0.533	–	–	–	–	188 (1)
Self‐esteem	Ex	0.3441	−0.330, 1.018	–	–	–	–	145 (1)

Abbreviations: 95% CI, 95% lower and upper confidence intervals; Corr, correlational study; Ex, experimental design; *I* ^2^, statistics for the proportion of variation across studies attributed to heterogeneity; *k*, number of studies; *N*, total sample size; PI, prediction interval; *Q*, Cochran's *Q* statistic for heterogeneity and *χ* ^2^ test; *τ* ^2^, variance of true effect size.

***<0.001; *<0.05.

*Source*: Authors.

In the meta‐analyses conducted on both correlational and experimental studies, a significant relationship between exposure to hate content and depressive symptoms in individuals has been observed. The synthesis of five correlational studies reveals a modest to moderate association (*d*_corr_ = 0.27; 95% CI = 0.011–0.53; *p* < 0.05), while the synthesis of two experimental studies indicates a strong causal relationship between this exposure and depressive symptoms (*d*_exp_ = 1.105; 95% CI = 0.797, 1.423; *p* < 0.01). Furthermore, exposure to hate content is found to be weakly but significantly linked to a decrease in life satisfaction among the exposed individuals (*d*_corr_ = −0.186; 95% CI = −0.279, −0.093; *p* < 0.01), as well as an increase in perceived societal fear, specifically regarding whether society is more characterized by fear today compared to before a terrorist attack (*d*_corr_ = −0.206; 95%CI = 0.147, 0.264; *p* < 0.01). However, the conducted meta‐analyses do not provide sufficient evidence to establish a definitive association or causal relationship between this exposure and emotional responses such as aversion to the content or content‐related anxiety. Likewise, they do not yield a significant causal connection between exposure to hate content and negative emotional reactions toward individuals with protected characteristics or an increase in anxiety in their presence. Except for the limited number of studies per meta‐analysis, these results are quite reliable in light of the risk of bias analysis.

##### 5.3.5.1 Outcomes not included in meta‐analysis

Three additional psychological effects were identified based on single studies, which could not be included in a meta‐analysis due to insufficient data. A large non‐individual‐based study (Saha et al., 2019) examined the relationship between exposure to hate content and the expression of stress online, finding a moderate and highly significant effect size (*d*_nbi_ = 0.4; IC del 95% = 0.391, 0.409; *p* < 0.001). In contrast, the impact of exposure to hate content on positive emotional reactions toward groups with protected characteristics did not show significant effects, nor did it affect the self‐esteem of the exposed individuals.

### 5.4 Heterogeneity

Heterogeneity is present in practically all the meta‐analyses performed (Tables 5, 6, 7, 8, 9). However, following the proposition put forth by Borenstein (2019), the reliability of the data necessitates a minimum of 10 studies per meta‐analysis. We conducted meta‐analyses that count between two and nine studies each. Although recognizing the inherent limitations, we proceeded with these analyses and considered their outcomes as exploratory in nature. Borenstein (2019) also proposes that the prediction interval is the only valid indicator that allows us to assess the magnitude of data dispersion in a meta‐analysis. Thus, as far as possible, we will also report these results.

Within the dimension “Attitudinal changes,” the *Q*‐value is significant for Attitudes that support hate content, Explicit negative attitudes (experimental and correlational studies), and Negative stereotypes. This was calculated in three of the six meta‐analyses of this dimension, given that it requires at least two studies to be performed. This interval shows us that there is a high dispersion of results in Explicit negative attitudes (PI_Exp_ = −1.054, 1.882), Negative stereotypes (PI = −0.796, 1.364), and Positive attitudes (PI = −1.904, 1.45).

Within the dimension “Intergroup dynamics,” the meta‐analysis examining the variable “Perceived discrimination” reveals significant heterogeneity (*Q*‐value = 17.997; *p* < 0.01), accompanied by a considerable dispersion (PI = −7.636, 8.32). The variable “Intergroup trust” does not appear to exhibit any significant variations across the available data. However, given the limited number of studies available for analysis (only two), it becomes challenging to ascertain the extent of the dispersion accurately.

In terms of “Interpersonal behavior,” all meta‐analyses conducted, except for the analysis pertaining to “Hate crimes,” exhibit a significant *Q*‐value, thereby suggesting the existence of heterogeneity among the studies encompassed within these analyses. Regrettably, the assessment of the extent of dispersion via the prediction interval is not feasible in this scenario, as no meta‐analysis involving three or more studies has been conducted.

Regarding the dimension of “psychological effects,” six of the nine meta‐analyses yielded a significant Q‐value (Aversion_Exp_, Aversion_Corr_, Content Anxiety, Depression_Corr_, Negative Emotional Reaction, Relational Anxiety), indicating the presence of heterogeneity. Notably, in four of these meta‐analyses, a considerable dispersion between studies was observed, as indicated by the prediction intervals (Aversion_Corr_ = −9.253,8.607; Content Anxiety = −2.735,3.126; Depression_Corr_ = −0.676,1.217; Negative Emotional Reaction = −1.213,1.24). Among the meta‐analyses with significant results, only those associated with experimental studies on Depression and correlational studies on Life Satisfaction and Social Fear displayed no evidence of heterogeneity. The meta‐analysis concerning depressive symptoms lacked sufficient studies to establish a prediction interval, while the prediction interval for Life Satisfaction was not provided due to an estimated zero between‐study variance. Consequently, the meta‐analysis on social fear stands out as the only one with a confirmed low dispersion among study results. This can be attributed to the fact that all five studies included in this meta‐analysis involved the same international comparison.

#### 5.4.1 Meta‐regression

To elucidate the sources of heterogeneity within the conducted meta‐analyses, two distinct methods were employed: meta‐regression and moderation analysis. Meta‐regression was employed with two continuous variables, namely, the proportion of male participants within the sample and the average age of the sample. The information for each study on these variables can be seen in Table 2. Meta‐regression was exclusively conducted when the meta‐analysis consisted of a minimum of five individual studies. Each variable was individually incorporated into separate models, employing univariate analysis. Consequently, the meta‐regression could only be performed in the case of four meta‐analyses. In some instances, certain moderator variables could not be analyzed due to insufficient information available in the studies or because the variable was a constant. The resulting findings are documented in Table 10.

**Table 10:** Meta‐regression for the percentage of men and mean age of the samples.

Dimension/outome	*k*	*B* (SE)	95% CI	*p*
*Attitudinal changes*				
Explicit negative attitudes_Exp_				
% Males	7	−0.0135 (0.014)	−0.0409, 0.014	0.3363
Average age	8	−0.0368 (0.0225)	−0.0808, 0.0072	0.1014
Negative stereotypes_Exp_				
% Males	8	−0.0155 (0.0124)	−0.0399, 0.0088	0.2118
Average age	8	0.0195, (0.0169)	−0.0137, 0.0526	0.2495
*Psychological effects*				
Depression_Corr_				
% Males	5	−0.0045 (0.0393)	−0.0815, 0.0725	0.9092
Average Age	5	0.0705 (0.0397)	−0.0073, 0.1482	0.0756
Social Fear_corr_				
% Males	5	−0.0226 (0.0091)	−0.0404, −0.0047	0.0132
Average Age	5	−0.0000 (0.0101)	−0.0199, 0.0198	0.9961

*Source*: Authors.

The average age of the samples included in this study seems to have an influence on the effect size obtained in the case of the correlational studies that evaluated the association between exposure to hate and depressive symptoms (*B* = 0.0705; *p* < 0.1). Similarly, the percentage of men in the samples influences the results of the meta‐analysis on the association between exposure to hate and Social Fear (*B* = ‐0.0226; *p* < 0.05). None of the other variables included in these meta‐regressions seem to have a significant influence on the results obtained in these four meta‐analyses.

#### 5.4.2 Moderation analysis

In the context of the moderation analysis, three categorical variables were utilized, namely: the nature of the media involved, the geographical region, and the classification of individuals exposed. The media types examined in this review encompassed traditional media, political propaganda, and social media on the Internet. Owing to the predominant focus on Western countries in the selected studies, a categorization was implemented to distinguish between studies based on samples from the United States and those from other countries. Moreover, the exposed individuals were categorized as belonging to either a targeted minority group frequently subjected to hate content or a nonspecific group. As in the case of meta‐regression, we considered the possibility of using moderation analysis only for meta‐analyses including at least five studies. The results can be found in Tables 11, 12, 13.

**Table 11:** Moderation analysis concerning the geographical region of samples.

Outcome	Region	*k*	*d*	95% CI	QBetween	*p*
Explicit negative attitudes_Exp_	USA	3	0.711	−0.065, 1.487	0.825	0.364
	Others	5	0.259	−0.332, 0.850		
Negative stereotypes_Exp_	USA	2	0.027	−0.618,0.672	0.796	0.372
	Others	7	0.362	0.008,0.717		
Depression_Corr_	USA	7	–	–	–	–
	Others	–	–	–	–	–
Social Fear_corr_	USA	1	0.297	0.249, 0.349	4.4401	0.0351
	Others	4	0.179	0.130, 0.228		

*Source*: Authors.

**Table 12:** Moderation analysis concerning the type of media involved.

Outcome	Media type	*k*	*d*	95% CI	QBetween	*p*
Explicit negative attitudes_Exp_	Propaganda	4	0.365	−0.332, 1.062	1.317	0.518
	Social media	1	−0.172	−1.532, 1.188		
	Traditional media	3	0.736	−8.639, 1.558		
Negative stereotypes_Exp_	Propaganda	3	0.477	0.018, 0.936	1.665	0.438
	Social media	4	0.241	−0.291, 0.773		
	Traditional media	2	−0.039	−0.689, 0.612		
Depression_Corr_	Propaganda	–	–	–	–	–
	Social media	5	–	–		
	Traditional media	–	–	–		
Social Fear_corr_	Propaganda	–	–	–	–	–
	Social media	5	–	–	–	–
	Traditional media	–	–	–		

*Source*: Authors.

**Table 13:** Moderation analysis concerning the type of individuals exposed.

Outcome	Minority group	*k*	*d*	95% CI	QBetween	*p*
Explicit negative attitudes_Exp_	No	7	0,444	0.020, 0.908	0.089	0.766
	Yes	1	0.245	−0.980, 1.470		
Negative stereotypes_Exp_	No	8	0.300	−0.036, 0.636	0.072	0.788
	Yes	1	0.160	−0.803, 1.124		
Depression_Corr_	No	2	0.184	−0.253, 0,620	0.238	0.623
	Yes	3	0.327	−0.047, 0.700		
Social Fear_corr_	No	5	–	–	–	–
	Yes	–	–	–		

*Source*: Authors.

The region of origin appears to significantly affect the association between exposure to hate and social fear (*Q* = 4.44, *p* < 0.05). Specifically, the effect size of this relationship is greater in the United States (*d* = 0.297) than in other regions (*d* = 0.179). Given that there is only one sample from the United States, these findings should be interpreted cautiously. Additionally, none of the other categorical variables in these moderation analyses seem to affect the effect size in the meta‐analyses investigated.

Overall, the analyses we have performed to explore the sources of heterogeneity have been inconclusive, which may be due to two reasons: first, the number of studies included in each meta‐analysis, as indicated at the beginning of this section, is not sufficient to have full confidence in these results and second, there are probably other underlying variables in our studies not identified in this review that could explain this heterogeneity. Following Borenstein (2019), the presence of heterogeneity does not necessarily invalidate the results of these meta‐analyses, but it is an important indicator to take into account in further studies.

### 5.5 Sensitivity analysis

We synthesized experimental and quasi‐experimental studies in the same meta‐analysis. We could then have performed a sensitivity analysis to determine whether quasi‐experimental studies influenced effect size or indicators of heterogeneity. However, the meta‐analyses that included both quasi‐experimental and experimental studies (Resistance to hate speech and Depression), were each conducted with only two studies so sensitivity analysis could not be performed.

We therefore decided to use the “One study removed” technique offered by the Comprehensive Meta‐Analysis (CMA) software to identify and investigate possible outliers within each meta‐analysis (see Table 14). Like meta‐analyses, one‐study‐removed analyses are more robust when more studies are included. Therefore, we restricted these analyses to meta‐analyses with the largest number of studies, specifically the meta‐analyses of experimental studies on the outcomes “Explicit Negative Attitudes” and “Negative Stereotypes.” For each outcome, the decision as to which study should be removed from the analysis was based first on the study whose relative weight was most important and second, on the study with the largest difference in effect size from the whole meta‐analysis.

**Table 14:** Sensitivity analysis.

Outcome		*d*	95% CI	*Q* value	Removed study
Explicit negative attitudes (Exp)	All studies	0.414	0.005,0.824	109.583**	
	One study removed	0.189	−0.143, 0.520	11.265**	Steele et al., 2015
Negative stereotypes (Exp)	All studies	0.28	−0.018, 0.586	75.783**	
	One study removed	0.148	−0.006, 0.352	25.699**	Matthes & Schmuck, 2017

*Source*: Authors.

** *p* < 0.01.

In these two outcomes, the weight of each study was balanced so that the study presenting results more distant from the whole meta‐analysis was removed. Even when important changes in the effect size are observed descriptively, particularly in the first study, there are no important variations in the confidence interval, so it is not considered that the removed studies significantly change the effect size of the meta‐analyses. In both cases, heterogeneity persists when the studies are removed (*Q*_Explicit negative attitudes_ = 11.265, *p* < 0.05; *Q*_Negative stereotypes_ = 25.699, *p* < 0.01).

### 5.6 Publication biases

To assess publication bias, we have applied two methods: the Trim‐and‐Fill and the Egger's regression. Borenstein (2019) recommends that this analysis be performed only when there are at least 10 studies included in a meta‐analysis. None of our meta‐analyses reaches this number. However, we will exploratorily address the publication risks in the two meta‐analyses where the largest number of studies are concentrated, namely the meta‐analyses addressing the experimental impact of hate exposure on Explicit negative attitudes (eight studies) and Negative stereotypes (nine studies).

Table 15 shows the results of this analysis. In both cases, there is a potential publication bias in these meta‐analyses which should include four additional studies according to the Trim‐and‐Fill method. This analysis is confirmed by Egger's regression, which is significant for the meta‐analysis of the influence of exposure on Explicit negative attitudes (*p* < 0.05) and slightly significant for the meta‐analysis of the influence of exposure on Negative stereotypes (*p* < 0.1).

**Table 15:** Publication biases of meta‐analyses on Explicit negative attitudes and Negative stereotypes.

Outcome	Trim‐and‐Fill						Egger's regression
	*d*	k	Imputed studies	*d* adjusted	95% CI	Q	*Β*0 (*p* value)
Explicit negative attitudes	0.414	8	4	−0.11384	−0.54593, 0.31824	265.224	6.34 (0.02)
Negative stereotypes	0.28	9	4	−0.01586	−0.33974, 0.30802	171.426	3.92 (0.07)

*Source*: Authors.

## 6 DISCUSSION

### 6.1 Summary of main results

The primary aim of this systematic review was to thoroughly examine and summarize the impact of exposure to or consumption of hate content in the media on individuals or groups who are subjected to it. We aimed to give practitioners and researchers a better understanding of how hateful rhetoric impacts targeted individuals and communities. These analyses show that exposure to hate is significantly associated with negative changes in attitudes toward others, more negative intergroup dynamics, a higher likelihood of interpersonal violence, and increased psychological distress.

In terms of attitude changes, the meta‐analyses demonstrate moderate and significant effects between exposure to hate content and the emergence of explicit negative attitudes toward individuals or groups with protected characteristics. This observation is reinforced by both correlational and experimental studies. The synthesis of the eight experimental studies further solidifies the idea that exposure to hate content may cause the formation of explicit negative attitudes.

The two additional findings align with the preceding observation. While the effect sizes are small and marginally significant, they suggest that exposure to hate content might contribute to the development of negative stereotypes regarding individuals with protected characteristics. Additionally, it could impede the promotion of positive attitudes toward them. Inversely, exposure to hate content does not appear to influence the development of favorable attitudes toward the content itself, nor does it seem to escalate support for political violence.

In line with the existing literature, our meta‐analyses confirm a large effect size between exposure to hate and victimization. This implies that individuals exposed to hate online are often the targets of violent behaviors offline. Moreover, moderate effect sizes were found for the relation between exposure to hate online and the online perpetration of hate speech, as well as the development of violent behaviors offline. On the other hand, there is no evidence to suggest that exposure to hate online fosters resistance behaviors among individuals who are frequently exposed to it. In essence, exposure to hate speech online and in the media is linked to both violent offline victimization and online perpetration of hate.

The observation that exposure to hate online is not associated with resistance behaviors but rather with hate perpetration is pre‐occupying in terms of increased polarization of the social space, and use of incrementally confrontational techniques and confirms that hate breeds hate. What has been referred to in the literature as “hate contagion” (Gallacher, 2021; Spörlein & Schlueter, 2021) is also confirmed by our meta‐analyses that show moderate and significant effect sizes of the spreading capacity of hate comments on other comments posted online. Comments identified as hate speech tend to generate new comments of the same nature. In contrast, comments identified as hate speech do not appear to have an impact on the occurrence of hate crimes in specific geographical areas.

In terms of psychological consequences, our results show that hate online impacts individual's psychological well‐being. Experimental studies indicate a large and significant effect size in relation to the development of depressive symptoms due to exposure. Additionally, a small effect size is observed concerning the link between exposure and reduced life satisfaction, as well as the perception of increased social fear in society. Conversely, exposure to hate speech does not seem to generate or be linked to the development of negative emotions related to its content.

Importantly, the impact of exposure to hate online does not depend on (or is not influenced by) the evaluation that exposed individuals make of its content, especially those individuals who do not come from a minority group. We refer particularly to the attitudes that support the content and the aversion and anxiety that this content may generate, where the studies that evaluated these outcomes mainly exposed Nonspecific Groups and consequently, non‐minority groups. Exposure to hate appears to impact personal emotional reactions (or symptoms) and attitude changes toward others, independently from the emotional and cognitive reactions to the hate content itself. Precisely, individuals exposed to hate do not seem to judge or emotionally react positively or negatively to the content they are observing, in ways different from other online content. Conversely, exposure to hate does demonstrate a transformation in the attitudes toward and perceptions of individuals targeted by hate, particularly minorities, as well as more severe symptomatic emotions that arise in those exposed. It can thus be concluded that, whether a person rejects or accepts the hateful content, the negative impact of hate on their perceptions and emotions remains significant. Given the findings supporting the contagion effect of hate online, this result is of utmost importance and has important implications for awareness campaigns, prevention programs, and interventions that we will discuss in the next sections.

Previous findings are based solely on the experiences of individuals or, alternatively, on the analysis of individual comments. The impact that exposure to hate can have on groups or communities is rarely addressed in the extant literature. When these dimensions are addressed, as in the case of intergroup dynamics, they are also analyzed based on the experiences of individuals. The extensive literature on these issues also tends to approach them from the perspective of vicarious victimization, which, as noted in the “The Problem” section, again corresponds to the individual experience of people who belong to the targeted groups but who have not been directly victimized by these acts. Vicarious victimization would demonstrate the impact of exposure to hate on broader populations. Studying the “group” or “community” level then seems to present additional difficulties for researchers or to be of less interest to them. Either way, it is an important gap in the existing literature.

In terms of intergroup dynamics, we found a small yet significant effect size, indicating that exposure to hate diminishes trust between groups, particularly between groups that are the target of such discourse and the general population. However, no significant effect size was found between this exposure and the perception of discrimination, particularly among minority groups. It is difficult to explain why exposure to hate does not increase the perception of discrimination in individuals who are frequently subjected to it. It could be interpreted that conceptually as well as in terms of persons' daily lived experience, hate, and discrimination may represent separate categories in the included studies. Hate may reflect discursive, cognitive, and emotional collective experiences online and in the media, while discrimination may refer to behavioral experiences in an individual's daily social interactions. Additionally, it is possible that for these minority groups, hate has become so commonplace and ingrained in their everyday lives that it almost goes unnoticed, as it has become an expected part of their public experience. It could also be argued that hate may be connected to real‐life experiences that are perceived as more severe, such as hate incidents and hate crimes. More studies are needed to address this issue.

Finally, our review did not find significant differences in relation to the type of media through which these messages were transmitted. This means that hate speech has relatively similar impacts whether it is transmitted through social media or traditional media. This may be because most of the studies that used traditional media were also based on their online content, and the number of studies included in each meta‐analysis was too small to detect a clear difference.

One element that could not be addressed in this systematic review is the relationship between exposure to hate content and violent extremism. We found significant associations at the behavioral level (victimization, perpetration of hate speech, violent behavior), but experimental studies could not prove that exposure to hate increases or decreases support for political violence. The only outcome that associated exposure to hate with the development of extreme political views (Extreme views) could not be included in a meta‐analysis because it had only one study. This relationship is extremely important, as there are many conceptual and empirical gray areas between the two phenomena that in many cases lead to confusion between the two (Nesbitt, 2021; Taylor, 2019). At least 5% of acts listed as hate crimes in the FBI database have also been listed as terrorist acts in the Global Database of Terrorism (Asal et al., 2012). Many studies do in fact associate hate‐based acts with violent right‐wing extremism and white supremacism and it is well documented that terrorist acts have led to an increase in hateful comments on social media (Asal et al., 2012, 2013; Olteanu et al., 2018). This confusion may be in part due to a lack of clear definitions, but most likely due to the intersecting nature of these two phenomena and the difficulty researchers face in measuring radicalization and violent extremism. It is not possible to conclude from this systematic review that exposure to hate may be associated with the development of violent extremism and at least one of the meta‐analyses shows that exposure to hate does not necessarily have an impact on support for political violence. This being said, the link between hate and violent extremism presents many blind spots that need further elucidation by research.

Given that many of these changes are of causal nature, we can confidently conclude that the results of this systematic review demonstrate that exposure to hate online and in traditional media has adverse impacts on individuals, communities, and society more generally.

### 6.2 Overall completeness and applicability of evidence

Although the field of hate studies began to develop in the 1990s, this systematic review shows that a significant portion of the studies retained, particularly those focusing on the role of media, including social media and online targeting, have been published since 2015, indicating a recent surge in research in these areas. Despite its recency, having included both experimental and correlational studies, as well as non‐individual‐based studies is evidence of the completeness of this study.

Being a recent field, there are still many gray areas in which solid evidence on the relationship between exposure to hate and its potential consequences has not yet been established. Proof of this is that at least 19 outcomes identified in the literature could not be part of a meta‐analysis because they did not have the minimum number of studies to do so. For example, no outcome associated with the “political beliefs” dimension could be included in a meta‐analysis. The fact that all but two of the meta‐analyses had only five or fewer studies is also another example. This last element prevented a more in‐depth analysis of the possible contextual and moderating variables that could influence the effect sizes. This is the case with the types of exposure initially deemed important for this systematic review (“exposure to,” “active search for,” and “participation”), but which could not be analyzed in depth because almost all the studies are based solely on simple exposure to this content. More studies are needed that would allow us to explore these unevaluated outcomes and moderators with greater certainty.

Despite the completeness of this systematic review, the considerable search effort in the gray literature, as well as the double screening of scientific articles, it is possible that certain relevant articles have not been identified through this process. However, it is unlikely that these non‐identified studies would substantially change the evidence presented in this systematic review. The sensitivity analyses performed showed that, even if some types of studies were eliminated, the results did not change substantially.

Thus, the knowledge acquired through this systematic review applies to the reality of the field today with the limits it has.

### 6.3 Quality of the evidence

No study that was evaluated with our assessment tool was removed from the systematic review. This means that the 55 included studies are of sufficient quality for their evidence to be reliable. In the experimental studies, participants had high adherence to the experimental conditions and the vast majority, therefore, contributed to most of the results. The correlational studies used consistent, valid, and reliable instruments to measure exposure and outcomes, which were derived from well‐defined variables. As with the experimental studies, the results of the studies were clearly complete.

There are, however, some areas in which these studies are less developed. Only a few experimental studies clearly explained how the process of randomization of participants to each of the conditions occurred. It was also unclear whether the groups associated with each of the conditions were comparable at the baseline. The non‐randomized studies were generally quite robust except for the representativeness of their samples in relation to their target population.

The use of information from social media is a topic that deserves a separate discussion, as it faces several methodological and ethical challenges (Ahmed et al., 2017; Beninger et al., 2014). In methodological terms, online comments may, for example, come from a few users who have multiple accounts or from automated bots producing many comments, which affects the representativeness of the data (Beninger et al., 2014; Sinnenberg et al., 2017). The data collected may often be based on the same platform and the same time period, such as Twitter data, compounded by potential errors in machine learning algorithms that are used to analyze large data sets. In addition, many users interact differently on social networks than in real life (Beninger et al., 2014), which may undermine the validity of this type of study. From an ethical point of view, the main concern is the use of data without the consent of individuals (Ahmed et al., 2017). This field of study is, however, growing and can hardly be overlooked given the importance of social media today. These studies have the advantage of analyzing the natural language of the people involved in these events and the context of these settings and thus exploring sources of information that have not been accessible to scientific research before. The studies included in this review address these methodological issues in one way or another.

### 6.4 Limitations and potential biases

Despite the promising results shown by this systematic review, it has certain limitations that may bias the interpretation of these results. These limits are mainly associated with the scope of this systematic review, the large number of outcomes identified, the limited number of studies retained, and the state of the current literature on the subject. In other words, the diversity of potential outcomes of hate exposure that have been investigated has been, in proportional terms, more important than the number of studies that have addressed these issues.

As mentioned above, we were able to identify 43 different outcomes, 19 of which could not be meta‐analyzed because they had only one study. Most of these meta‐analyses were also performed with two or three studies and most of them showed heterogeneity. Heterogeneity, however, is not conclusive because it depends mainly on the number of studies included. It is likely that by increasing the number of studies per outcome this heterogeneity will disappear or can be explained by moderating variables, which will allow refining the theoretical construction of these outcomes.

Publication bias cannot be minimized, especially considering the unresolved debate between Anderson and Ferguson around the impact on children of exposure to violent media content (Anderson & Bushman, 2002; Anderson et al., 2010; Ferguson & Beresin, 2017; Ferguson, 2015). That is, the tendency to publish positive results or where an impact is demonstrated, which may be hiding research where these results have not been conclusive. In the 2 meta‐analyses where publication bias was analyzed, a potential bias was found. Therefore, we advise caution when interpreting the results of the meta‐analyses.

## 7 CONCLUSION

### 7.1 Implications for practice and policy

This systematic review helps to consolidate an intuition that many policymakers and practitioners express about the impact of hate online and in traditional media. Our study reveals a significant association between exposure to hate content through media and its negative impact on exposed individuals and groups. We consider the findings of this systematic review of utmost importance for their implications for policy design, prevention, and intervention.

The core of this problem lies in the ability of hate speech to spread either through comments that reproduce the same content or through facilitating a negative and biased perception of individuals and groups with protected characteristics. This implies that hate speech has the potential to trivialize and normalize prejudices that, in turn, can harm targeted communities through negative stereotypes, forms of racism and discrimination, and ultimately through acts of violence if we consider that this exposure is related to both victimization and perpetration of hate speech and violent behavior by exposed individuals.

Hate speech alone does not elicit negative or positive emotional or cognitive responses to the content itself from exposed non‐minority individuals. What this means is that people who view this content do not express more shock or disgust, nor do they rate the content as more disgusting or stimulating, as compared to any other type of content found in the media.

The fact that hate content does not seem to elicit repulsion may speak to the desensitization and normalization of such hate in the online environment. This may also explain the ease with which this content spreads as it may be considered harmless. This may also explain why in some cases people may consider the regulation of this content to be exaggerated and violating their freedom of expression. In this perspective, public policies and initiatives that aim to censor or counter hate may be perceived as illegitimate or less effective in preventing its negative consequences. It is also likely that campaigns or programs focusing solely on the content of hate speech may have mitigated effects in bringing about significant changes.

On the other hand, regardless of its evaluation by viewers, exposure to online hate still significantly influences the attitudes toward individuals targeted by hate and can erode trust between communities. This means that on the societal scale, exposure to hate can foster social polarization by fostering us vs them frameworks, hostility between different social groups, as well as social fear. Consequently, divisive political discourses emphasizing differences between groups and threats to the majority groups' identity are likely to crystalize the negative attitudes further and erode trust between groups. Policy, prevention, and intervention programs will then need to focus on addressing stereotypes and negative attitudes toward minorities, as well as re‐establishing trust between minority communities and the majority.

Our systematic review also shows that exposure to hate increases the likelihood of online and offline violence and results in more severe symptomatic emotions that arise in those exposed. In terms of policy, these results support the importance of regulating hate online as well as providing accessible psychological or mental health services to those who are exposed to hate online. For practitioners working on the ground in prevention, our results suggest that programs aiming to develop responsible behavior on and optimal use of the online space, resilience to hate online and offline, abilities to engage in constructive social dialogue and debates, and prevention of violence offline may merit to be developed and evaluated. For practitioners working in the intervention space with perpetrators and targeted individuals, our results support the relevance of providing psychosocial and mental health services accessible to accompany perpetrators on the path of desistance from violence as well as support targets on their path of resilience to hate and mental well‐being.

Finally, despite the scope of the results obtained in this systematic review, the impacts of exposure to hate cannot be reduced to the limited number of studies that were found in this process. Hate exposure may have other impacts on individuals and communities that researchers have not yet been able to identify or imagine.

### 7.2 Implications for research

This study has several implications for future research. This systematic review synthesizes the current evidence on the effects or correlations of exposure to hate online and in traditional media, but it also acknowledges the limitations of the meta‐analytic approach and the need for further research to complement its results and to examine potential moderators of the observed outcomes. One of the underexplored variables in this literature is political beliefs, whose outcomes could not be included in our analysis due to insufficient data. Another area that warrants more attention is the link between violent extremism and hate speech. As we noted earlier, the boundaries between these two phenomena are quite blurry and there is some indication of how terrorist attacks can trigger the dissemination of hate speech. However, we have little knowledge of how hate speech can influence the formation of extremist or political views.

At the same time, even if experimental studies allow us to answer the question about the causal impact of exposure to hate on violence and well‐being, there are very few longitudinal studies that have explored with greater certainty, and in the medium and long term, the consequences that this exposure can have on individuals and communities. Of particular interest may be the exploration through longitudinal studies of the trajectories of hate‐based perpetration.

Finally, there is still a long way to go about social media‐based studies. Despite the methodological problems already mentioned, it is quite evident that it is not possible to avoid these sources of information, given their importance in our contemporary life and particularly concerning the proliferation of hate speech. It is the type of information that can probably most easily report impacts on a broad social scale, as long as it is understood that these studies have their particularities and that their conclusions should be applied exclusively to the contexts from which they emerge. To best deliver such studies, it is necessary to address the methodological and ethical challenges that this type of research poses.

## CONTRIBUTIONS OF AUTHORS

- Content: Pablo Madriaza, Ghayda Hassan, Sébastien Brouillette‐Alarie.
- Systematic review methods: Pablo Madriaza, Eugene Borokhovski, Ghayda Hassan,
- Statistical analysis: Pablo Madriaza, Aoudou Njingouo Mounchingam.
- Information retrieval: David Pickup, Loïc Durocher‐Corfa.
- Scientific Writing and Editing: Pablo Madriaza, Ghayda Hassan.
- Data Coding: Pablo Madriaza, Loïc Durocher‐Corfa, Aoudou Njingouo Mounchingam, Sabrina Paillé.

## DECLARATIONS OF INTEREST

The research team has no potential conflict of interest for this review.

## PLANS FOR UPDATING THE REVIEW

Dr. Pablo Madriaza and Dr. Ghayda Hassan and their research team will be responsible for updating this review, every 5 years after the publication date.

## SOURCES OF SUPPORT

The research team has received funding from Public Safety Canada and approval to move forward with the review.

## Supporting information

Supporting information.

## Footnotes

1 Available at https://www.campbellcollaboration.org/research-resources/effect-size-calculator.html.

2 We decided in this case to include the minimum geographical unit reported, which in this case were cities (Relia et al., 2019) and municipalities (Müller & Schwarz, 2021). Another study with comparable characteristics, conducted by Nguyen et al. (2021) employed counties in the United States as the unit of analysis. Regrettably, this study could not be incorporated into the present meta‐analysis due to its utilization of risk ratios, which are not compatible with the studies included in this analysis.
