# Supplementary material for: Exposure to hate in online and traditional media: A systematic review and meta‐analysis of the impact of this exposure on individuals and communities
Source: Campbell Syst Rev. 2025 Jan 16;21(1):e70018. doi: 10.1002/cl2.70018 (PMC11736891; doi:10.1002/cl2.70018)
Supplement: Supplementary file 1 — Supporting information. [file CL2-21-e70018-s001.docx]

# Online supplements

## Appendix A: Search strings

**PsycINFO**

((hatred or hate or dangerous or fanatic* or prejudic* or intoleran* or bias or violen* or negative or stigmat* or discriminat* or bigot* or hostil* or "desire to destroy" or "desire to damage" or oppress* or abuse or abusi* or "desire to kill" or humiliat* or intimidat* or terrori*) adj2 (crime* or speech or incident* or conduct or act or acts or abuse* or vilif* or language or harass* or word* or express* or comment* or defam* or slur* or troll* or flaming or "flame war" or incite* or insult* or "call* for" or derog*)).ab,hw,id,mh,ot,sh,ti.

(radical* or indoctrinat* or fundamentalis* or "homegrown terror" or terror* or eco-terror* or "Al Qaida" or ISIS or anti-capitalis* or extremis* or supremacis* or nationali* or "homegrown threat*" or Jihad* or "white power" or "neo-nazi" or "right wing" or "right-wing" or "left wing" or "left-wing" or nativis* or "anti-immigra*" or "ecological violence" or "anti-capitalis*" or islamophob* or "alt-right" or antifa* or incel or homophob* or discriminati* or transphobi* or antisemiti* or anti-semiti* or mysogyn* or xenophob* or homophob* or anifemini* or racis* or stereotyp* or sexis* or gender or "sex* identit*" or LGBT*).ab,hw,id,mh,ot,sh,ti.

(website* or "information system*" or "electronic communication*" or online or "social media" or "world wide web" or "web 2.0" or internet or virtual or cyber or website* or digital or "computer media*" or bebo or facebook or flickr or foursquare or friendster or hulu or instagram or linkedin or meetup or pinterest or reddit or snapchat or tumblr or xing or twitter or yelp or youtube or tiktok or photolog or telegram or whatsapp or messenger or twitch or discord or gab or "chat room*" or "online forum*" or "discussion forum*" or "videogame communit*" or "dark web" or "dark net" or "deep web" or "computer crime" or "anonymity network*" or "image board*" or 4chan or 8chan or meme* or media or communication* or telecommunication* or radio or television or newspaper* or news or cinema or movie* or journalis* or theatre or music or video*).ab.

1 and 2 and 3

limit 4 to human

**Web of Science**

TOPIC: ((hatred OR hate OR dangerous OR fanatic* OR prejudic* OR intoleran* OR bias OR violen* OR negative OR stigmat* OR discriminat* OR bigot* OR hostil* OR "desire to destroy" OR "desire to damage" OR oppress* OR abuse OR abusi* OR "desire to kill" OR humiliat* OR intimidat* OR terrori*) NEAR/2 (crime* OR speech OR incident* OR conduct OR act OR acts OR abuse* OR vilif* OR language OR harass* OR word* OR express* OR comment* OR defam* OR slur* OR troll* OR flaming OR "flame war" OR incite* OR insult* OR "call* for" OR derog*)) AND TOPIC: (radical* OR indoctrinat* OR fundamentalis* OR "homegrown terror" OR terror* OR eco-terror* OR "Al Qaida" OR ISIS OR anti-capitalis* OR extremis* OR supremacis* OR nationali* OR "homegrown threat*" OR Jihad* OR "white power" OR "neo-nazi" OR "right wing" OR "right-wing" OR "left wing" OR "left-wing" OR nativis* OR "anti-immigra*" OR "ecological violence" OR "anti-capitalis*" OR islamophob* OR "alt-right" OR antifa* OR incel OR homophob* OR discriminati* OR transphobi* OR antisemiti* OR anti-semiti* OR mysogyn* OR xenophob* OR homophob* OR anifemini* OR racis* OR stereotyp* OR sexis* OR gender OR "sex* identit*" OR LGBT*) AND TOPIC: (website* OR "information system*" OR "electronic communication*" OR online OR "social media" OR "world wide web" OR "web 2.0" OR internet OR virtual OR cyber OR website* OR digital OR "computer media*" OR bebo OR facebook OR flickr OR foursquare OR friendster OR hulu OR instagram OR linkedin OR meetup OR pinterest OR reddit OR snapchat OR tumblr OR xing OR twitter OR yelp OR youtube OR tiktok OR photolog OR telegram OR whatsapp OR messenger OR twitch OR discord OR gab OR "chat room*" OR "online forum*" OR "discussion forum*" OR "videogame communit*" OR "dark web" OR "dark net" OR "deep web" OR "computer crime" OR "anonymity network*" OR "image board*" OR 4chan OR 8chan OR meme* OR media OR communication* OR telecommunication* OR radio OR television OR newspaper* OR news OR cinema OR movie* OR journalis* OR theatre OR music OR video*)

Refined by: DOCUMENT TYPES: ( ARTICLE OR REVIEW OR BOOK CHAPTER OR DATA PAPER OR PROCEEDINGS PAPER OR REPRINT OR EARLY ACCESS )

Timespan: All years. Indexes: SCI-EXPANDED, SSCI, A&HCI, CPCI-S, CPCI-SSH, ESCI.

**EBSCO Databases**

Searched separately: Academic Search Complete, Communication Abstracts, Communication & Mass Media Complete, Criminal Justice Abstracts, Education Source, ERIC, Political Science Complete, SocINDEX

S1: TI ( (hatred OR hate OR dangerous OR fanatic* OR prejudic* OR intoleran* OR bias OR violen* OR negative OR stigmat* OR discriminat* OR bigot* OR hostil* OR "desire to destroy" OR "desire to damage" OR oppress* OR abuse OR abusi* OR "desire to kill" OR humiliat* OR intimidat* OR terrori*) N2 (crime* OR speech OR incident* OR conduct OR act OR acts OR abuse* OR vilif* OR language OR harass* OR word* OR express* OR comment* OR defam* OR slur* OR troll* OR flaming OR "flame war" OR incite* OR insult* OR "call* for" OR derog*) ) OR AB ( (hatred OR hate OR dangerous OR fanatic* OR prejudic* OR intoleran* OR bias OR violen* OR negative OR stigmat* OR discriminat* OR bigot* OR hostil* OR "desire to destroy" OR "desire to damage" OR oppress* OR abuse OR abusi* OR "desire to kill" OR humiliat* OR intimidat* OR terrori*) N2 (crime* OR speech OR incident* OR conduct OR act OR acts OR abuse* OR vilif* OR language OR harass* OR word* OR express* OR comment* OR defam* OR slur* OR troll* OR flaming OR "flame war" OR incite* OR insult* OR "call* for" OR derog*) ) OR KW ( (hatred OR hate OR dangerous OR fanatic* OR prejudic* OR intoleran* OR bias OR violen* OR negative OR stigmat* OR discriminat* OR bigot* OR hostil* OR "desire to destroy" OR "desire to damage" OR oppress* OR abuse OR abusi* OR "desire to kill" OR humiliat* OR intimidat* OR terrori*) N2 (crime* OR speech OR incident* OR conduct OR act OR acts OR abuse* OR vilif* OR language OR harass* OR word* OR express* OR comment* OR defam* OR slur* OR troll* OR flaming OR "flame war" OR incite* OR insult* OR "call* for" OR derog*) ) OR SU ( (hatred OR hate OR dangerous OR fanatic* OR prejudic* OR intoleran* OR bias OR violen* OR negative OR stigmat* OR discriminat* OR bigot* OR hostil* OR "desire to destroy" OR "desire to damage" OR oppress* OR abuse OR abusi* OR "desire to kill" OR humiliat* OR intimidat* OR terrori*) N2 (crime* OR speech OR incident* OR conduct OR act OR acts OR abuse* OR vilif* OR language OR harass* OR word* OR express* OR comment* OR defam* OR slur* OR troll* OR flaming OR "flame war" OR incite* OR insult* OR "call* for" OR derog*) )

S2: TI ( (radical* OR indoctrinat* OR fundamentalis* OR "homegrown terror" OR terror* OR eco-terror* OR "Al Qaida" OR ISIS OR anti-capitalis* OR extremis* OR supremacis* OR nationali* OR "homegrown threat*" OR Jihad* OR "white power" OR "neo-nazi" OR "right wing" OR "right-wing" OR "left wing" OR "left-wing" OR nativis* OR "anti-immigra*" OR "ecological violence" OR "anti-capitalis*" OR islamophob* OR "alt-right" OR antifa* OR incel OR homophob* OR discriminati* OR transphobi* OR antisemiti* OR anti-semiti* OR mysogyn* OR xenophob* OR homophob* OR anifemini* OR racis* OR stereotyp* OR sexis* OR gender OR "sex* identit*" OR LGBT*) ) OR AB ( (radical* OR indoctrinat* OR fundamentalis* OR "homegrown terror" OR terror* OR eco-terror* OR "Al Qaida" OR ISIS OR anti-capitalis* OR extremis* OR supremacis* OR nationali* OR "homegrown threat*" OR Jihad* OR "white power" OR "neo-nazi" OR "right wing" OR "right-wing" OR "left wing" OR "left-wing" OR nativis* OR "anti-immigra*" OR "ecological violence" OR "anti-capitalis*" OR islamophob* OR "alt-right" OR antifa* OR incel OR homophob* OR discriminati* OR transphobi* OR antisemiti* OR anti-semiti* OR mysogyn* OR xenophob* OR homophob* OR anifemini* OR racis* OR stereotyp* OR sexis* OR gender OR "sex* identit*" OR LGBT*) ) OR KW ( (radical* OR indoctrinat* OR fundamentalis* OR "homegrown terror" OR terror* OR eco-terror* OR "Al Qaida" OR ISIS OR anti-capitalis* OR extremis* OR supremacis* OR nationali* OR "homegrown threat*" OR Jihad* OR "white power" OR "neo-nazi" OR "right wing" OR "right-wing" OR "left wing" OR "left-wing" OR nativis* OR "anti-immigra*" OR "ecological violence" OR "anti-capitalis*" OR islamophob* OR "alt-right" OR antifa* OR incel OR homophob* OR discriminati* OR transphobi* OR antisemiti* OR anti-semiti* OR mysogyn* OR xenophob* OR homophob* OR anifemini* OR racis* OR stereotyp* OR sexis* OR gender OR "sex* identit*" OR LGBT*) ) OR SU ( (radical* OR indoctrinat* OR fundamentalis* OR "homegrown terror" OR terror* OR eco-terror* OR "Al Qaida" OR ISIS OR anti-capitalis* OR extremis* OR supremacis* OR nationali* OR "homegrown threat*" OR Jihad* OR "white power" OR "neo-nazi" OR "right wing" OR "right-wing" OR "left wing" OR "left-wing" OR nativis* OR "anti-immigra*" OR "ecological violence" OR "anti-capitalis*" OR islamophob* OR "alt-right" OR antifa* OR incel OR homophob* OR discriminati* OR transphobi* OR antisemiti* OR anti-semiti* OR mysogyn* OR xenophob* OR homophob* OR anifemini* OR racis* OR stereotyp* OR sexis* OR gender OR "sex* identit*" OR LGBT*) )

S3: AB website* OR "information system*" OR "electronic communication*" OR online OR "social media" OR "world wide web" OR "web 2.0" OR internet OR virtual OR cyber OR website* OR digital OR "computer media*" OR bebo OR facebook OR flickr OR foursquare OR friendster OR hulu OR instagram OR linkedin OR meetup OR pinterest OR reddit OR snapchat OR tumblr OR xing OR twitter OR yelp OR youtube OR tiktok OR photolog OR telegram OR whatsapp OR messenger OR twitch OR discord OR gab OR "chat room*" OR "online forum*" OR "discussion forum*" OR "videogame communit*" OR "dark web" OR "dark net" OR "deep web" OR "computer crime" OR "anonymity network*" OR "image board*" OR 4chan OR 8chan OR meme* OR media OR communication* OR telecommunication* OR radio OR television OR newspaper* OR news OR cinema OR movie* OR journalis* OR theatre OR music OR video*

S4: S1 AND S2 AND S3

Filtered: Magazines, Newspapers, Trade Publications, Book Reviews

**ProQuest Databases**

Searched separately: ProQuest Central, ProQuest Dissertations & Theses Global, NCJRS, Sociological Abstracts (including Social Services Abstracts)

(NOFT(hatred OR hate OR dangerous OR fanatic* OR prejudic* OR intoleran* OR bias OR violen* OR negative OR stigmat* OR discriminat* OR bigot* OR hostil* OR "desire to destroy" OR "desire to damage" OR oppress* OR abuse OR abusi* OR "desire to kill" OR humiliat* OR intimidat* OR terrori*) ) NEAR/2 (NOFT(crime* OR speech OR incident* OR conduct OR act OR acts OR abuse* OR vilif* OR language OR harass* OR word* OR express* OR comment* OR defam* OR slur* OR troll* OR flaming OR "flame war" OR incite* OR insult* OR "call* for" OR derog*) )

AND

NOFT(radical* OR indoctrinat* OR fundamentalis* OR "homegrown terror" OR terror* OR eco-terror* OR "Al Qaida" OR ISIS OR anti-capitalis* OR extremis* OR supremacis* OR nationali* OR "homegrown threat*" OR Jihad* OR "white power" OR "neo-nazi" OR "right wing" OR "right-wing" OR "left wing" OR "left-wing" OR nativis* OR "anti-immigra*" OR "ecological violence" OR "anti-capitalis*" OR islamophob* OR "alt-right" OR antifa* OR incel OR homophob* OR discriminati* OR transphobi* OR antisemiti* OR anti-semiti* OR mysogyn* OR xenophob* OR homophob* OR anifemini* OR racis* OR stereotyp* OR sexis* OR gender OR "sex* identit*" OR LGBT*)

AND

AB(website* OR "information system*" OR "electronic communication*" OR online OR "social media" OR "world wide web" OR "web 2.0" OR internet OR virtual OR cyber OR website* OR digital OR "computer media*" OR bebo OR facebook OR flickr OR foursquare OR friendster OR hulu OR instagram OR linkedin OR meetup OR pinterest OR reddit OR snapchat OR tumblr OR xing OR twitter OR yelp OR youtube OR tiktok OR photolog OR telegram OR whatsapp OR messenger OR twitch OR discord OR gab OR "chat room*" OR "online forum*" OR "discussion forum*" OR "videogame communit*" OR "dark web" OR "dark net" OR "deep web" OR "computer crime" OR "anonymity network*" OR "image board*" OR 4chan OR 8chan OR meme* OR media OR communication* OR telecommunication* OR radio OR television OR newspaper* OR news OR cinema OR movie* OR journalis* OR theatre OR music OR video*)

**Medline (PubMed)**

S1: ((hatred or hate or dangerous or fanatic* or prejudic* or intoleran* or bias or violen* or negative or stigmat* or discriminat* or bigot* or hostil* or "desire to destroy" or "desire to damage" or oppress* or abuse or abusi* or "desire to kill" or humiliat* or intimidat* or terrori*) adj2 (crime* or speech or incident* or conduct or act or acts or abuse* or vilif* or language or harass* or word* or express* or comment* or defam* or slur* or troll* or flaming or "flame war" or incite* or insult* or "call* for" or derog*)).ab,hw,kf,kw,ot,sh,ti.

S2: (radical* or indoctrinat* or fundamentalis* or "homegrown terror" or terror* or eco-terror* or "Al Qaida" or ISIS or anti-capitalis* or extremis* or supremacis* or nationali* or "homegrown threat*" or Jihad* or "white power" or "neo-nazi" or "right wing" or "right-wing" or "left wing" or "left-wing" or nativis* or "anti-immigra*" or "ecological violence" or "anti-capitalis*" or islamophob* or "alt-right" or antifa* or incel or homophob* or discriminati* or transphobi* or antisemiti* or anti-semiti* or mysogyn* or xenophob* or homophob* or anifemini* or racis* or stereotyp* or sexis* or gender or "sex* identit*" or LGBT*).ab,hw,kf,kw,ot,sh,ti.

S3: (website* or "information system*" or "electronic communication*" or online or "social media" or "world wide web" or "web 2.0" or internet or virtual or cyber or website* or digital or "computer media*" or bebo or facebook or flickr or foursquare or friendster or hulu or instagram or linkedin or meetup or pinterest or reddit or snapchat or tumblr or xing or twitter or yelp or youtube or tiktok or photolog or telegram or whatsapp or messenger or twitch or discord or gab or "chat room*" or "online forum*" or "discussion forum*" or "videogame communit*" or "dark web" or "dark net" or "deep web" or "computer crime" or "anonymity network*" or "image board*" or 4chan or 8chan or meme* or media or communication* or telecommunication* or radio or television or newspaper* or news or cinema or movie* or journalis* or theatre or music or video*).ab.

S4: 1 and 2 and 3

S5: limit 4 to humans

## Appendix B: Document Coding Protocol

**Reference information**

1. Document ID
2. Study Title
3. Study Author(s)
4. Publication Year
5. Place published or accessed with URL
6. Reference Type
7. Journal article
8. Book/chapter
9. Government report: specify type and source
10. Organization report: specify type and source
11. Conference paper
12. Thesis/dissertation
13. Other
14. Coding References
15. Coder Name
16. Coding date
17. Coding duration
18. Coding issues/challenges

**Study details**

1. Country of Study:___
2. Document Language:____
3. Date of Research
4. Start:___
5. Finish:___
6. Peer reviewed
7. Yes
8. No
9. Funded research
10. Yes
11. If YES. Funding source:___
12. No
13. Unknown
14. Conflicts of interest:__
15. Ethical Issues:__

**Methodology**

1. Type of study:
2. Quantitative
3. Mixed Methodologies
4. Type of design:
   1. Experimental
   2. Quasi-experimental
   3. Correlational
5. Sample constitution procedure
6. Country/place of recruitment
7. Provide details on how the sample was constituted
8. Sample characteristics
9. Total sample size:
10. Sample gender:

i. Male

ii. Female

iii. Mixed

1. Sample Age:___
2. Sample socio-economic status:

i. Low

ii. Average

iii. High

iv. Mixed

v. Other:__

1. Sample ethnic racial group:__
2. Sample religious group:___
3. Groupe exposed: (minority = yes, no)
4. Other sample characteristic measured (if any):__
5. Source of hate speech measure:
6. Obtained from official data (government/police)
7. Self-reported
8. Peer-reported
9. Family-reported
10. Practitioner-reported
11. Experimental exposure
12. Other _________________________
13. Source of outcome measure:
14. Obtained from official data (government/police)
15. Self-reported
16. Peer-reported
17. Family-reported
18. Practitioner-reported
19. Other _________________________

18-A. Quantitative measures on link between exposure and outcome

1. Measures
2. Type of analyses conducted- provide details

18-B. Qualitative measures on link between exposure and outcome

1. Measures
2. Type of analyses conducted- provide details

**Independent Variable Details**

**Participants’ interaction with hate speech:**

1. Hate Speech
2. Target of hate speech
3. Perpetrator of hate speech
4. Type of hate speech
5. Exposure to hate
6. Content of exposure
7. Process/length of exposure
8. Frequency of exposure
9. Reasons for exposure
10. Other details of exposure
11. Active search of hate (when reported)
12. Details of active search
13. Content of active search
14. Process/length of active search
15. Frequency of active search
16. Reasons for active search
17. Participation
18. Details of participation
19. Content of participation
20. Process/length of participation
21. Frequency of participation
22. Reasons for participation

**Hate Speech medium**

1. Traditional media: (Traditional media, Mass media, Communication media, Telecommunications Media, Radio, Television, Newspaper, News, Cinema, Movies)

Describe:____

1. Online media: (Online media, Social Media, Web 1.0, Web 2.0, Internet, Virtual, Cyber, Site, Website, Online Social Networks, Online Community, Computer Mediated Communication, Information Systems, Communication Systems, Electronic Communication, online, bebo, facebook, flickr, foursquare, Friendster, Hulu, Instagram, linkedin, meetup, pinterest, reddit, snapchat, tumblr, xing, twitter, yelp, youtube

Describe:___

1. Political Propaganda

**Dependent variable details**

**Participants’ outcome after interaction with hate speech:**

1. Type of measured outcome:
2. Violent radicalization. Describe:
3. Mental health symptoms. Describe:
4. Emotions (negative or positive). Describe:
5. Attitudes. Describe:
6. Behavior. Describe:
7. Social fracturing (discrimination, ostracization). Describe:
8. Others. Describe:
9. Interaction variables measured
10. Trigger point or factors that explain outcome
11. Severity/level of measured outcome
12. Type (describe contextual variables that interact with IV and DV)
13. Other. Describe (gender, age, and the like)
14. Quantitative results on link between exposure and outcome
15. Statistical results- - provide details (description of results)
16. Benefits of measure used
17. Unexpected outcomes
18. Harms from study
19. Qualitative results on link between exposure and outcome
20. Summarize results
21. Benefits of measure used
22. Unexpected outcomes
23. Harms from study

**Authors’ Conclusion**

1. What did the authors conclude about the relationship?
2. Interaction with hate speech has measured outcomes on individuals, communities, and or societies
3. Interaction with hate speech does not have measured outcomes on individuals, communities, and or societies
4. Unclear/no conclusion stated by authors
5. Recommendations from authors
6. Yes. Describe: policy, research, practice
7. No
8. Study limitations
9. From authors. Describe:__
10. Identified by the team. Describe:__

## Appendix C: Mixed Methods Appraisal Tool (MMAT)

| **Study** | **Design** | **SC1** | **SC2** | **RCT1** | **RCT2** | **RCT3** | **RCT5** | **NR1** | **NR2** | **NR3** | **NR4** | **NR5** |
| --- | --- | --- | --- | --- | --- | --- | --- | --- | --- | --- | --- | --- |
| Anspach, 2021 | RCT | Y | Y | N | Y | Y | Y |  |  |  |  |  |
| Arendt et al., 2015 | RCT | Y | Y | N | I can't tell | Y | Y |  |  |  |  |  |
| Brinson, 2010a | RCT | Y | Y | Y | Y | Y | Y |  |  |  |  |  |
| Brinson, 2010b | RCT | Y | Y | Y | Y | Y | Y |  |  |  |  |  |
| Botan et al., 2020; Buturoiu & Corbu, 2020 | RCT | Y | Y | Y | I can't tell | Y | Y |  |  |  |  |  |
| Chavez et al., 2019 | RCT | Y | Y | Y | N | Y | Y |  |  |  |  |  |
| Hameleers, 2019 | RCT | Y | Y | N | I can't tell | Y | Y |  |  |  |  |  |
| Lee-Won et al., 2020 | RCT | Y | Y | I can't tell | I can't tell | Y | Y |  |  |  |  |  |
| Matthes & Schmuck, 2017 | RCT | Y | Y | N | I can't tell | Y | Y |  |  |  |  |  |
| Newman et al., 2021 | RCT | Y | Y | N | I can't tell | Y | Y |  |  |  |  |  |
| Obermaier et al., 2021 | RCT | Y | Y | N | Y | Y | Y |  |  |  |  |  |
| Rieger et al., 2013c | RCT | Y | Y | N | I can't tell | Y | Y |  |  |  |  |  |
| Schmuck & Matthes, 2017 | RCT | Y | Y | Y | Y | Y | Y |  |  |  |  |  |
| Schmuck & Matthes, 2019 | RCT | Y | Y | Y | Y | Y | Y |  |  |  |  |  |
| Schmuck & Tribastone, 2020 | RCT | Y | Y | Y | Y | Y | Y |  |  |  |  |  |
| Schmuck et al., 2017 | RCT | Y | Y | Y | Y | Y | Y |  |  |  |  |  |
| Shortland et al., 2022 | RCT | Y | Y | N | I can't tell | Y | Y |  |  |  |  |  |
| Soral et al., 2018b | RCT | Y | Y | N | I can't tell | Y | Y |  |  |  |  |  |
| Steele et al., 2015 | RCT | Y | Y | N | I can't tell | Y | Y |  |  |  |  |  |
| Velasco, 2016 | RCT | Y | Y | N | I can't tell | Y | Y |  |  |  |  |  |
| Weber et al., 2020 | RCT | Y | Y | N | I can't tell | Y | Y |  |  |  |  |  |
| Ziegele et al., 2018 | RCT | Y | Y | N | Y | Y | Y |  |  |  |  |  |
| Blaya & Audrin, 2019 | NRS | Y | Y |  |  |  |  | I can't tell | Y | Y | Y | Y |
| Cano et al., 2021 | NRS | Y | Y |  |  |  |  | Y | Y | Y | Y | Y |
| Costello et al., 2017 | NRS | Y | Y |  |  |  |  | Y | Y | Y | Y | Y |
| Costello et al., 2019 | NRS | Y | Y |  |  |  |  | Y | Y | Y | Y | Y |
| Dashti et al., 2015 | NRS | Y | Y |  |  |  |  | N | Y | I can't tell | N | Y |
| English et al., 2020 | NRS | Y | Y |  |  |  |  | Y | Y | Y | Y | Y |
| Gallacher, 2021 | NRS | Y | Y |  |  |  |  | Y | Y | Y | N | Y |
| Keipi et al., 2018a | NRS | Y | Y |  |  |  |  | Y | Y | Y | Y | Y |
| Keipi et al., 2018b | NRS | Y | Y |  |  |  |  | Y | Y | Y | Y | Y |
| Lee & Leets, 2002 | NRS | Y | Y |  |  |  |  | N | Y | Y | Y | Y |
| Müller & Schwarz, 2021 | NRS | Y | Y |  |  |  |  | Y | Y | Y | Y | Y |
| Näsi et al., 2015 | NRS | Y | Y |  |  |  |  | I can't tell | Y | Y | Y | Y |
| Nguyen et al., 2021 | NRS | Y | Y |  |  |  |  | Y | Y | Y | Y | Y |
| Oksanen et al., 2020a | NRS | Y | Y |  |  |  |  | I can't tell | Y | Y | Y | Y |
| Oksanen et al., 2020b | NRS | Y | Y |  |  |  |  | I can't tell | Y | Y | Y | Y |
| Oksanen et al., 2020c | NRS | Y | Y |  |  |  |  | I can't tell | Y | Y | Y | Y |
| Oksanen et al., 2020d | NRS | Y | Y |  |  |  |  | I can't tell | Y | Y | Y | Y |
| Oksanen et al., 2020e | NRS | Y | Y |  |  |  |  | I can't tell | Y | Y | Y | Y |
| Pauwels & Schils, 2016 | NRS | Y | Y |  |  |  |  | N | Y | Y | Y | Y |
| Räsänen et al., 2016 | NRS | Y | Y |  |  |  |  | Y | Y | Y | Y | Y |
| Relia et al., 2019 | NRS | Y | Y |  |  |  |  | Y | Y | Y | Y | Y |
| Saha et al., 2019 | NRS | Y | Y |  |  |  |  | Y | Y | Y | Y | Y |
| Soral et al., 2018a | NRS | Y | Y |  |  |  |  | Y | Y | Y | N | Y |
| Soral et al., 2018c | NRS | Y | Y |  |  |  |  | Y | Y | Y | N | Y |
| Spörlein & Schlueter, 2021 | NRS | Y | Y |  |  |  |  | I can't tell | Y | Y | Y | Y |
| TaeHyuk Keum & Hearns, 2022 | NRS | Y | Y |  |  |  |  | I can't tell | Y | Y | Y | Y |
| Tynes et al., 2008 | NRS | Y | Y |  |  |  |  | N | Y | Y | Y | Y |
| Tynes et al., 2014 | NRS | Y | Y |  |  |  |  | Y | Y | Y | Y | Y |
| Voigtländer & Voth, 2015 | NRS | Y | Y |  |  |  |  | Y | Y | Y | N | Y |
| Wachs et al., 2019, 2021 | NRS | Y | Y |  |  |  |  | N | Y | Y | Y | Y |
| Walker-Matthews, 1996 | NRS | Y | Y |  |  |  |  | I can't tell | Y | Y | Y | Y |
| Wojcieszak, 2010 | NRS | Y | Y |  |  |  |  | N | Y | Y | Y | Y |
| Ybarra et al., 2008 | NRS | Y | Y |  |  |  |  | Y | Y | Y | Y | Y |

**Design**

RCT = Random control trials

NRS = Nonrandomized studies

**MMAT indicators**

SC1 = Are there clear research questions?

SC2 = Do the collected data allow to address the research questions?

RCT1 = Is randomization appropriately performed?

RCT2 = Are the groups comparable at baseline?

RCT3 = Are there complete outcome data?

RCT5 = Did the participants adhere to the assigned intervention?

NRS1 = Are the participants representative of the target population?

NRS2 = Are measurements appropriate regarding both the outcome and intervention (or exposure)?

NRS3 = Are there complete outcome data?

NRS4 = Are the confounders accounted for in the design and analysis?

NRS5 = During the study period, is the intervention administered (or exposure occurred) as intended?

## Appendix D: Partial and complete effects

| **Dimension** | **Outcome** | **Design** | **Effect size** |
| --- | --- | --- | --- |
| Attitudinal changes | Attitudes that support hate content | Ex | Complete effect |
|  | Explicit negative attitudes | Ex | Partial effect |
|  |  | Corr | Partial effect |
|  | Negative stereotypes | Ex | Partial effect |
|  | Positive attitudes | Ex | Complete effect |
|  | Support for political violence | Ex | Complete effect |
| Intergroup dynamics | Intergroup trust | Ex | Complete effect |
|  | Perceived discrimination | Ex | Complete effect |
| Interpersonal behavior | Exogroup discrimination intent | Corr | Complete effect |
|  | Online perpetration | Corr | Partial effect |
|  | Online victimization | Corr | Partial effect |
|  | Violent behavior | Corr | Partial effect |
|  | Resistance to hate speech | Ex | Complete effect |
|  | Hate contagion | NBI | Partial effect |
|  | Hate crimes | NBI | Partial effect |
| Psychological effects | Aversion | Ex | Complete effect |
|  |  | Corr | Complete effect |
|  | Content Anxiety | Corr | Partial effect |
|  | Depression | Ex | Complete effect |
|  |  | Corr | Partial effect |
|  | Negative emotional reaction toward groups with protected characteristics | Ex | Partial effect |
|  | Relational anxiety | Ex | Partial effect |
|  | Satisfaction with life | Corr | Partial effect |
|  | Social Fear | Corr | Partial effect |
